# Global, regional and national estimates of the burden of childhood asthma attributable to NO_2_ exposure for 204 countries and territories from 1990 to 2023: a Global Burden of Disease study 2023

**DOI:** 10.1016/j.eclinm.2025.103580

**Published:** 2025-11-01

**Authors:** Katrin Burkart, Sarah Wozniak, Susan Anenberg, Ana Pereda, Nora Gilbertson, Charlie Ashbaugh, Daniel Goldberg, Perry Hystad, Gaige H. Kerr, Susan A. McLaughlin, Arash Mohegh, Michael Brauer

**Affiliations:** a Institute for Health Metrics and Evaluation, University of Washington, Seattle, WA, USA; b Milken Institute School of Public Health, George Washington University, Washington DC, USA; c College of Health, Oregon State University, Corvallis, OR, USA; d School of Population and Public Health, The University of British Columbia, Vancouver, BC, Canada

**Keywords:** Burden of disease, Pediatric asthma, Nitrogen dioxide

## Abstract

**Background:**

Asthma, a chronic lung condition characterised by inflammation and airway constriction, has been associated with nitrogen dioxide (NO_2_) exposure, an association that particularly impacts children. Our study rigorously assessed this relationship and estimated the global burden of childhood asthma attributable to NO_2_ exposure in 204 countries and territories from 1990 to 2023.

**Methods:**

We systematically reviewed epidemiological studies evaluating NO_2_'s long-term impact on childhood asthma. Using burden of proof meta-regression methods that account for bias by adjusting for study-design covariates and quantify remaining unexplained between-study heterogeneity to incorporate into uncertainty, we estimated the relative risk of childhood asthma occurring as a function of NO_2_ exposure. From this, we computed global and country-specific population attributable fractions (PAFs) – i.e., the proportional change in asthma risk that would occur if NO_2_ exposure were reduced to a theoretical minimum exposure level range of 4.6–6.2 ppb. We applied PAFs to data from the 2023 Global Burden of Disease Study (GBD) to derive the asthma burden attributable to NO_2_ exposure in children and youths under 20 years old. Burden of proof methods allowed us to further compute risk–outcome metrics quantifying the magnitude of the NO_2_–asthma association and its strength of supporting evidence.

**Findings:**

We identified a total of 27 cohort studies, spanning 12 countries, primarily in Europe and high-income North America, with some studies from Asia (China and Japan). A meta-regression log-linear risk curve of these studies produced a summary RR of 1.05 (95% UI 0.99–1.12) per 5 ppb NO_2_ and Egger's regression indicated significant publication bias. We estimated a global PAF of 4.67% (95% uncertainty interval [UI]: −3.75 to 20.6; all UIs reported in this study are inclusive of between-study heterogeneity except where noted), yielding 233,000 (−250,000–956,000) years lived with disability (YLDs) attributable to NO_2_ globally in 2023. GBD 2023 ranked NO_2_ seventh among environmental risk factors contributing to YLDs in children for all causes and third for childhood asthma YLDs. Attributable burden estimates and trends varied significantly by GBD super-region. While NO_2_-attributable childhood asthma burden has declined substantially since 1990 in the high-income and central Europe, eastern Europe, and central Asia super-regions, NO_2_ remains a prominent environmental risk factor in these two super-regions, ranked third in both super-regions for paediatric asthma YLDs in 2023, contributing 57,100 (−63 600 to 242,000) and 6570 (−7050 to 30,200) YLDs, respectively. In South Asia, NO_2_ ranks second as risk factor for pediatric asthma contributing to 20,100 (−17 600, 105,000) YLDs in 2023.

**Interpretation:**

NO_2_ pollution remains a top environmental risk for paediatric health, necessitating policy interventions targeting NO_2_ pollution, especially in high-income locations and urban areas.

**Funding:**

The research described in this article was conducted in part under contract with the 10.13039/100001160Health Effects Institute (HEI), an organization jointly funded by the 10.13039/100000139United States Environmental Protection Agency (EPA) and certain motor vehicle and engine manufacturers, grant number 4977/20-11. The contents of this article do not necessarily reflect the views of HEI or its sponsors, nor do they necessarily reflect the views and policies of the EPA or motor vehicle and engine manufacturers. Additional funding for this study was received by the Gates foundation, grant no. OPP1152504 for MB, KB, and SW. SA, DG, AM and GHK were supported by 10.13039/100000104NASA grant no. 80NSSC21K0511 and SA and GHK 10.13039/100000002NIH grant no. P20ES036775.

**Research in context**

**Evidence before this study**

Several recent epidemiological studies indicate a correlation between elevated levels of nitrogen dioxide (NO_2_), a gaseous component of ambient air, and increased childhood asthma incidence and prevalence. However, the strength and reliability of this association remains uncertain due to methodological variations and inconsistent outcomes across studies. Limited systematic assessments have been conducted to address this heterogeneity across studies.

**Added value of this study**

This study builds upon the global scope and internal coherence established by previous Global Burden of Disease (GBD) investigations, offering a robust framework for a comprehensive evaluation of NO_2_-related asthma burden and facilitating cross-comparisons with other GBD risk factors. Our research significantly enhances understanding of the significance of the burden of paediatric asthma that is attributable to NO_2_ exposure by applying the following analysis tools to rigorously interrogate the existing data. First, we systematically assessed all available evidence concerning the long-term association between NO_2_ exposure and childhood asthma risk, utilizing the newly developed burden of proof framework. This approach enabled us to incorporate study-specific covariates to account for known variation in input study design characteristics, then quantify remaining between-study heterogeneity and adjust uncertainty intervals of the meta-regressed relative risk (RR) estimates to address unexplained variability. Second, we used RR estimates to compute sex- and age-specific population attributable fractions (PAFs) reflecting asthma risk attributable to NO_2_ exposure, NO_2_-attributable years lived with disability (YLDs), and NO_2_-attributable incident asthma cases for individuals aged 0 to 19 across 204 countries and territories from 1990 to 2023. Third, we conducted a decomposition analysis to elucidate the factors influencing trends in NO_2_-attributable asthma burden over time, including contributions from population growth, demographic ageing, changes in asthma incidence, and NO_2_ exposure levels. Complementing these investigations, we assessed the impact of the reduced pollution during the COVID-19 pandemic on the asthma burden attributable to NO_2_ by calculating PAFs under a hypothetical counterfactual exposure scenario.

**Implications of all the available evidence**

Our study underscores the significant impact of NO_2_ pollution on YLDs for children globally. Notably, GBD 2023 ranked NO_2_ as the seventh leading environmental risk factor worldwide for all-cause YLDs in children and the second leading risk factor for childhood asthma-related YLDs. Globally, approximately 4.67% of asthma YLDs in children were attributable to NO_2_ exposure in 2023. Furthermore, we observed substantial variations in NO_2_-attributable burden and trends across GBD super-regions. For instance, while the high-income and central Europe, eastern Europe, and central Asia super-regions have witnessed significant decreases in NO_2_-attributable burden since 1990, NO_2_ remained among the top-ranked environmental risk factors contributing to all-cause and asthma-related paediatric YLDs in these areas in 2023. In contrast, although NO_2_ was ranked lower (eighth) as an environmental risk factor contributing to paediatric YLDs in sub-Saharan Africa, the burden of childhood asthma is on the rise in this region due to increasing NO_2_ exposure coupled with population growth. Our findings also highlight that NO_2_-attributable asthma burden is predominantly an urban phenomenon. Notably, the COVID-19 pandemic led to a reduction in NO_2_-attributable PAFs for childhood asthma, especially in the high-income super-regions, likely due to reduced traffic density in the large urban centres of these regions.

## Introduction

Recent epidemiological evidence suggests that nitrogen dioxide (NO_2_), a key component of ambient air pollution and a common marker for traffic-related air pollution, especially in urban areas, is associated with the development of asthma.^1^, ^2^, ^3^, ^4^, ^5^, ^6^, ^7^ Asthma is a chronic inflammatory disease characterized by bronchial hyper-responsiveness leading to airway inflammation and constriction.^8^ Studies of airway response to NO_2_ exposure indicate that NO_2_ amplifies inflammatory response in airways via oxidative stress.^9^ Some studies highlight increasing neutrophil^9^ activity as a source of airway inflammation, while others suggest heightened eosinophil^10^^,^^11^ activity and sensitivity to allergens.^10^^,^^12^ Studies of the biological mechanism of NO_2_-exacerbated inflammation have prompted population-based studies on the relationship between NO_2_ and asthma, particularly childhood asthma.^1^^,^^4^^,^^13^ Though previous research has underlined variability in study design and findings, growing supports a negative impact of NO_2_ on childhood asthma.^1^^,^^14^

In 2023, asthma accounted for 5.23 million (95% uncertainty interval [UI] 3.33–7.56 million) years lived with disability (YLDs) globally among individuals under 20 years.^15^ Asthma burden is highest in high-income areas like North America, western Europe, and Australasia, but it is also high in Latin America and the Caribbean. Paediatric asthma YLD rates have notably surged in recent years in high-income North America, from 505.5 (321.72–739.28) per 100,000 in 1990 to 646.46 (407.89–968.32) per 100,000 in 2024. While asthma YLD rates for children have dropped since 1990 in most regions, there have been moderate increases in central Europe, eastern Europe, and central Asia; Southeast Asia, East Asia, and Oceania and the high-income super-region since the mid-2000s.^15^ These trends underscore the relevance of examining NO_2_ as a risk factor for childhood asthma.

Here, we explore NO_2_ as a risk factor for childhood asthma morbidity. Firstly, we systematically gathered all relevant case–control and cohort studies on the link between long-term NO_2_ exposure and childhood asthma risk, and extracted relevant data to conduct a meta-analysis and regression. We estimated the summary relative risk function using burden of proof methodology, which is widely used within the Global Burden of Disease Study (GBD).^16^ The Burden of Proof framework offers a new, data-driven approach to objectively assess the strength and certainty of associations between risk factors and health outcomes. To account for heterogeneity across input studies, burden of proof methods incorporates covariates to account for potentially systematic variation in study design characteristics and also quantify and incorporate estimates of remaining unexplained between-study heterogeneity. Additionally, the burden of proof framework employs a novel risk-scoring framework to conservatively evaluate the magnitude of the risk-outcome relationship and the strength of the evidence supporting it. By introducing the Burden of Proof Risk Function (BPRF), it estimates the minimum level of risk consistent with available data—essentially the most conservative risk that can still be justified. This methodology enables standardized comparisons across different risk–outcome pairs and translates findings into an intuitive star rating to aid communication and guide policy decisions.

Secondly, we computed sex-specific population attributable fractions (PAFs) to estimate asthma risk attributable to NO_2_ for individuals aged 0 to 19 across 204 countries and territories from 1990 to 2023 based on the comparative risk factor assessment framework.^17^^,^^18^ We examined spatial and temporal patterns of the NO_2_-attributable asthma burden and estimated associated YLDs and incident asthma cases. Thirdly, we analysed the factors influencing trends in NO_2_-attributable asthma burden over this period, examining population growth, ageing, asthma incidence, and NO_2_ exposure. Finally, we assessed the impact of the COVID-19 pandemic on NO_2_-attributable asthma burden by calculating PAFs for a counterfactual exposure scenario.

## Methods

### Systematic review

Following the GBD protocol,^17^ we identified the most recent peer-reviewed systematic review and meta-analyses adhering to PRISMA guidelines^19^ on long-term NO_2_ exposure and paediatric asthma. From Khreis et al.'s^1^ comprehensive 2017 meta-analysis on traffic-related air pollution's effects on childhood asthma, we extracted 20 of the 31 NO_2_-related components studies. To ensure consistency, we excluded 11 studies that overlapped with multiple publications from the same cohort, retaining only estimates for the longest follow-up time. Additionally, we searched PubMed and Embase for updates until December 2019 to relevant literature using specific keywords. Our inclusion and exclusion criteria matched those of Khreis et al.'s^1^ meta-analysis and our search was limited to the English language. We updated the literature search through June 2025 and identified seven additional studies. These studies were not incorporated into the current burden estimates, as they were published after the cutoff date for inclusion into the GBD. Furthermore, we extracted information about study design characteristics that may introduce systematic bias. We excluded cross-sectional studies due to their less robust design. Details of search strings, input data, inclusion/exclusion criteria, and data extraction methods are provided in the Appendix. Figure S1 presents the search strategy and results in a PRISMA diagram format.

### Meta-analysis

We analysed the collected data from the systematic review through a meta-regression to estimate the relative risk (RR) of childhood asthma as a function of NO_2_ exposure using the burden of proof framework, which employs ensemble spline methods to capture the potentially non-linear shape of the RR function.^16^^,^^20^ For this, we meta-regressed log-transformed observations, ie RR, OR or HR, incorporating the inverse observation variances to give more weight to larger studies with smaller uncertainties and improve the certainty of final estimates. We first tested fitting a cubic spline with three interior knots, randomly placed by an ensemble process, on the dataset. To avoid overestimation in the upper range of the data—where measurement points were sparse—we evaluated the effect of different priors on the upper segments of the curve. However, our data did not suggest a nonlinear form of the exposure-response relationship and we opted to model a log-linear meta-analytic summary.

The burden of proof framework allowed us to generate robust estimates of the association between NO_2_ and paediatric asthma while addressing heterogeneous input data through two key components.^16^^,^^20^ First, we used automated covariate selection capacity to identify study-level covariates representing known variation in study design characteristics that significantly affected the meta-regression model. In our analysis, we considered several key covariates: whether the study focused on a specific subpopulation; whether exposure was assessed at the individual or population level; whether exposure was measured pre- and postnatal, and at baseline or at multiple time points; whether the outcome was self-reported or medically verified; whether prevalence or incidence was assessed, whether the study was a randomized controlled trial; and the extent to which known confounders (e.g., age, sex, education, parental asthma/allergies, and particulate matter pollution) were accounted for. Additionally, we adjusted for loss to follow-up, follow-up duration, and the type of outcome measure used (i.e., RR, HR, or OR). While we tested for statistical significance, our choice of covariates was primarily guided by clinical relevance and their potential to capture meaningful differences in exposure or outcome assessment, study design, and analytical approach. A detailed overview of all covariates and their definitions is provided in Table S1. Second, we incorporated remaining unexplained between-study heterogeneity into predictions of uncertainty of the pooled effect size and generated 500 final predictions of the effect size at 5 ppb NO_2_ exposure for use in burden calculation. All UIs reported in this study are inclusive of between-study heterogeneity except where noted. We incorporated Fisher information into predictions of between-study heterogeneity to correct for data-sparsity and ensure we were not underestimating heterogeneity due to limited data. We also detected and trimmed 10% of input observations during model fitting to render the model robust to outliers.^20^

### Population attributable fraction and burden estimation

We calculated NO_2_-attributable burden on the 0.0083 × 0.0083° (approximately 1 × 1 km) resolution using the comparative risk assessment framework (CRA) and the following inputs: the summary RR estimate derived from our meta-analysis, estimates of annual average surface-level NO_2_ exposure, a theoretical minimum risk exposure level (TMREL), and burden estimates (expressed in YLDs) for childhood asthma.^15^ For GBD 2023, annual average NO_2_ surface concentrations were estimated at a 50 m × 50 m spatial resolution in five-year increments from 1990 to 2005, and annually from 2005 to 2023. Estimates of NO_2_ surface concentration were based on products from Larkin et al.,^21^^,^^22^ which integrates satellite remote-sensing data with land-use and meteorological variables. A detailed description of methods is available in the GBD risk factor publication.^23^

To propagate model uncertainty in burden estimation, we generated 500 draws of each grid cell's concentration. We assumed complete correlation between deviation estimates due to lack of information on posterior correlation relationships.

We used a counterfactual NO_2_ exposure level, or TMREL, of a uniform distribution between 4.6 and 6.2 ppb. This represents the means of the minimum and 5th percentiles for the five RR literature input studies with the lowest reported exposure values. This approach to TMREL estimation captures uncertainty in characterizing the dose–response relationship at low exposure levels and reflects that an NO_2_ concentration of 0 is implausible.

PAFs quantify the proportion of asthma burden attributed to NO_2_ pollution and reflect the burden that can hypothetically be mitigated by reducing NO_2_ pollution to the TMREL. Previously, Mohegh and colleagues^24^ determined that a spatial resolution of 0.0083 × 0.0083° was optimal for NO_2_-attributable burden calculations, balancing both computational efficiency and concentration resolution.^24^ Accordingly, we calculated PAFs for individuals <20 years of age for 5-year increments at this resolution. We generated 500 draws of all intermediate inputs and estimates using the formula

$$PAF=AF*{pop}_{exposed}=\frac{RR\;-\;1}{RR}*1$$

Let AF represent the attributable fraction, and ${pop}_{exposed}$ denote the proportion of the population exposed. In the case of air pollution, where the entire population is exposed, ${pop}_{exposed}\;equals\;1\text{.}$

Using population data from the WorldPop Database, we generated population-weighted aggregates of PAFs for the GBD subnational and national locations.^25^ We then interpolated PAFs for the complete time series from 1990 to 2023 using the Piecewise Cubic Hermite Interpolating Polynomial (PCHIP) method. Due to a lack of evidence on NO_2_'s association with asthma mortality, these PAFs contributed only to asthma morbidity. To estimate NO_2_-attributable burden, we multiplied the resulting PAFs with age-, sex-, year-, and location-specific prevalence and YLD estimates from GBD 2023.^15^ Uncertainty estimates are generated by propagating modelling uncertainties from exposure and RR estimates based on 500 draws. Sources of uncertainty that our approach cannot incorporate stem from measurement and selection bias as well as model specification bias.

To assess the impact of the COVID-19 pandemic-related mobility reductions on NO_2_ emissions and therefore NO_2_-attributable burden, we estimated childhood asthma burden attributable to a counterfactual level of NO_2_ exposure. This counterfactual, calculated as the mean of annual ambient NO_2_ exposure levels in 2017, 2018, and 2019, was chosen to represent NO_2_ exposure in the absence of transport and industry changes due to the COVID-19 pandemic. We calculated counterfactual PAFs using the methods described above.

### Risk-outcome scores

As a complement to RR, PAF, and attributable burden estimates, the burden of proof framework generates risk score metrics – the burden of proof risk function (BPRF) and associated risk-outcome score (ROS) and star ratings – that provide a conservative estimate, for a given risk–outcome pair, of both the change in health outcome associated with risk factor exposure and the strength of the evidence for this association. Risk-score metrics provide an empirical measure of the association and evidence strength for risk-outcome pairs across GBD risk factors and are therefore useful for standardized comparison.^16^ For harmful risk factors, including NO_2_ pollution, the BPRF is represented by the 5th percentile RR curve, inclusive of uncertainty estimates incorporating between-study heterogeneity, closest to a null RR value of 1. The ROS is a summary measure calculated by averaging the signed value of the log BPRF, across the data-rich domain between the 15th to 85th percentiles of the input data risk exposure distribution (NO_2_ = 9.99–56.00 ppb).^16^ For ease of comparison across risk–outcome pairs, ROSs are mapped onto a star-rating system with one to five stars, with more stars corresponding to a greater effect size and/or stronger evidence for the association. We additionally tested for publication bias in the input data using the Egger's Regression strategy.^26^

### Role of the funding sources

The funders of the study had no role in study design, data collection, data analysis, data interpretation, or writing of the report. The corresponding author had full access to all the data in the study and had final responsibility for the decision to submit for publication.

## Results

### Systematic review and meta-analysis

We identified a total of 27 cohort studies examining the relationship between ambient NO_2_ pollution and childhood asthma incidence or prevalence. These studies span 20 locations and the years 1987–2019 (for a full list of all studies, see Appendix). Together, these studies’ NO_2_ exposure distributions spanned a range of 9.9–56 ppb, which covers a fraction of the global exposure range of <1.0–140 ppb as estimated by Anenberg and colleagues.^27^ Input studies assessing RR were conducted in 12 countries, primarily in Europe and high-income North America, with some studies from Asia (China and Japan). A log-linear meta-analysis of these studies produced a summary RR of 1.05 (95% UI with gamma 0.99–1.12; 95% UI without gamma 1.04–1.06) per 5 ppb NO_2_. Three study-specific covariates were found to explain between-study variability related to study-level characteristics and were included in the analysis: 1) self-reported asthma diagnosis, 2) risk measure (RR versus OR/HR), outcome measure (prevalence versus incidence). Coefficients indicate greater effect sizes for hazard ratios (HRs) and odds ratios (ORs) compared to risk ratios (RRs), with stronger associations observed when asthma is self-reported and when prevalence rather than incidence is assessed. We detected a weak but not significant association between observation residuals and their standard errors (p-value = 0.06), Egger mean = 0.173, Egger SD = 0.111), suggesting the potential for publication bias. We calculated an ROS of −0.03, which indicates that, based on currently available evidence, the relationship between NO_2_ exposure and childhood asthma received a rating of one star, interpreted in the burden of proof framework as weak evidence of an association. While it is possible that the introduction of new evidence would change this assessment, a one-star rating still warrants inclusion into the GBD. Table 1 provides an overview over RR estimates with and without in-between study heterogeneity *y* and other BoP parameters. Risk estimates that account for inter-study heterogeneity incorporate variations in effect estimates across studies to generate uncertainty ranges. For details about risk score metrics, see the Appendix and Zheng et al.^16^

**Table 1:** Strength of the evidence for the relationship between NO_2_ and paediatric asthma and RR increase per 5 ppb.

Health outcome	RR (95% UI without *y*)	RR (95% UI with *y*)	ROS	Star rating	Publication bias	No of studies	Selected bias covariates	Pair in GBD
Pediatric asthma	1.05 (1.04–1.06)	1.05 (0.99–1.12)	−0.03	1 star	Y	27	Risk measure, self-reported outcome	Y

RR = Relative risk; *y* = in-between study heterogeneity; ROS = Risk outcome score.

### Burden estimates

**Fig. 1:**
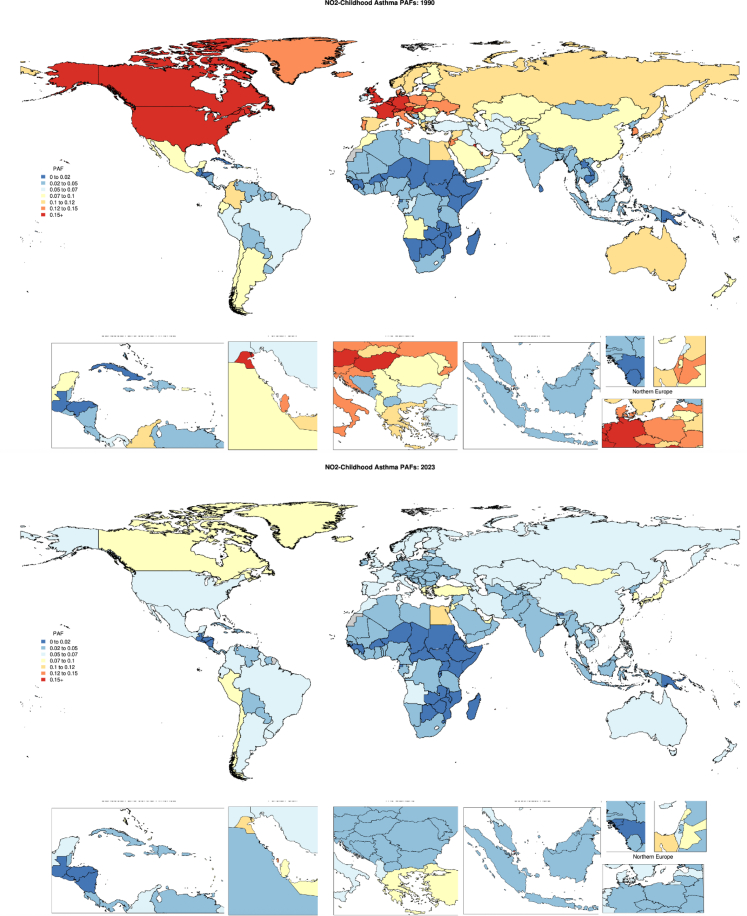
**Population attributable fraction of asthma YLDs attributable to NO_2_ for individuals <20 years of age in 1990 (upper) and 2023 (lower)**.

**Fig. 2:**
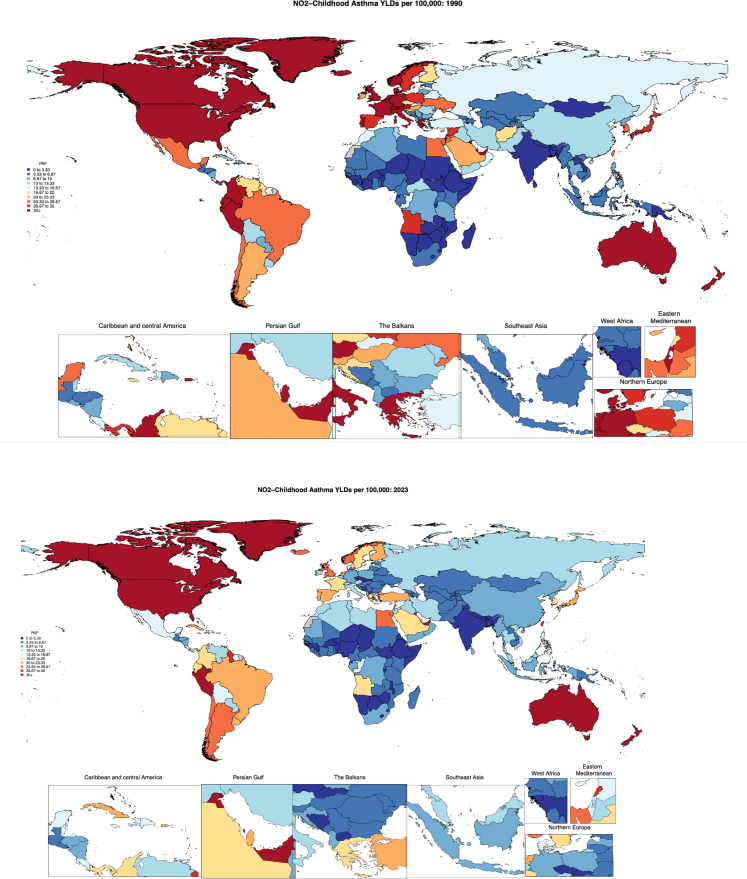
**Rate per 100,000 of asthma YLDs attributable to NO_2_ for individuals <20 years of age in 1990 (upper) and 2023 (lower)**.

In 2023, NO_2_ contributed 233,000 (95% UI –250,000 to 956,000) YLDs associated with paediatric asthma globally for children under 20 years of age (Table 2). The high-income and north Africa and Middle East super-region experienced the greatest percentage of NO_2_-attributable paediatric asthma YLDs across super-regions, with 6.45% (−5.69, 28.1) and 6.30% (−5.01, 25.2) paediatric asthma YLDs attributable to NO_2_ pollution, respectively (Table 2, Fig. 1) in 2023. The Southeast Asia, East Asia, and Oceania super-region also observed considerable percentages of NO_2_-attributable paediatric asthma burden, with 5.30% (−4.32, 23.5) of childhood asthma YLDs attributable to NO_2_. NO_2_ pollution contributed the lowest proportion of paediatric asthma YLDs in sub-Saharan Africa, accounting for 2.34% (−1.67, 10.8) of YLDs (Table 2). Fig. 2 illustrates differences in national NO_2_-attributable paediatric asthma YLD rates per 100,000 in 2023, highlighting the relevance of NO_2_ as a risk factor in the Americas, especially Peru (37.41 [−44.36 to 133.66] NO_2_-attributable paediatric asthma YLDs per 100,000) the United States (30.16 [−33.65 to 137.04] YLDs per 100,000), and Canada (38.76 [−39.87 to 169.04]) as well as Australia (36.93 [−42.6 to 143.43]) and New Zealand (35.50 [−32.65 to 139.39]). Kuwait and the United Arab Emirates stand out with 55.95 (−73.24 to 211.29) and 39.68 (−42.39 to 161.94) NO_2_-attributable paedeatric asthma YLDs per 100,000, respectively.

**Table 2:** Asthma YLDs attributable to NO_2_ globally and by super-region for individuals <20 years of age.

Region	YLDs (Years lived with disability), number, 1990, <20 years with 95% UI	YLDs (Years lived with disability), number, 2023, <20 years with 95% UI	YLDs (Years lived with disability), percent, 1990, <20 years with 95% UI	YLDs (Years lived with disability), percent, 2023, <20 years with 95% UI	YLDs (Years lived with disability), rate (per 100,000), 1990, <20 years with 95% UI	YLDs (Years lived with disability), rate (per 100,000), 2023, <20 years with 95% UI
Global	334,000 (−438,000, 1,210,000)	233,000 (−250,000, 956,000)	8.14 (−8.24, 29.5)	4.67 (−3.75, 20.6)	14.8 (−19.4, 53.5)	8.71 (−9.36, 35.8)
Central Europe, Eastern Europe, and Central Asia	20,300 (−23,300, 73,400)	6570 (−7,050, 30,200)	10.9 (−8.99, 42.3)	4.77 (−3.69, 22.1)	14.6 (−16.7, 52.4)	6.36 (−6.82, 29.2)
High-income	151,000 (−208,000, 537,000)	57,100 (−63,600, 242,000)	16.6 (−19.3, 55.7)	6.45 (−5.69, 28.1)	59.2 (−81.5, 210)	23.9 (−26.5, 101)
Latin America and Caribbean	44,600 (−54,900, 165,000)	34,300 (−40,300, 134,000)	5.96 (−5.67, 22.7)	4.86 (−3.87, 19.7)	23.9 (−29.4, 88.7)	18.8 (−22.0, 73.5)
North Africa and Middle East	27,700 (−31,800, 106,000)	35,400 (−40,300, 139,000)	7.16 (−5.98, 27.7)	6.30 (−5.01, 25.2)	15.6 (−17.9, 59.4)	14.8 (−16.9, 58.3)
South Asia	11,000 (−11,100, 48,600)	20,100 (−17,600, 105,000)	4.24 (−3.39, 19.8)	3.83 (−2.81, 20.3)	2.06 (−2.08, 9.10)	2.89 (−2.54, 15.1)
Southeast Asia, East Asia, and Oceania	66,000 (−84,000, 275,000)	50,900 (−57,100, 215,000)	6.08 (−5.02, 24.8)	5.30 (−4.32, 23.5)	9.64 (−12.3, 40.2)	8.79 (−9.87, 37.2)
Sub-Saharan Africa	12,800 (−12,800, 52,300)	28,300 (−28,900, 118,000)	2.5 (−1.82, 11.2)	2.34 (−1.67, 10.8)	4.65 (−4.64, 18.9)	4.47 (−4.57, 18.7)

Globally, NO_2_ pollution ranked third out of all GBD risk factors contributing to childhood asthma YLDs in 2023 (with high BMI ranking second and secondhand smoke ranking first). In south Asia, NO_2_ ranked second while in all other super regions NO_2_ ranked third among risk factors contributing to paediatric asthma YLDs (Supplementary Material, Figure S4). With respect to all-cause paediatric YLDs, NO_2_ pollution ranked seventh among environmental risk factors in 2023 below water, sanitation, lead, hygiene, occupational ergonomic factors, and occupational injuries (Supplementary Material, Figure S5). NO_2_'s relative contribution to all-cause YLDs for children varied considerably by super-region, with NO_2_ ranked first in the high-income super-region and eighth in sub-Saharan Africa among environmental/occupational risk factors.

**Fig. 3:**
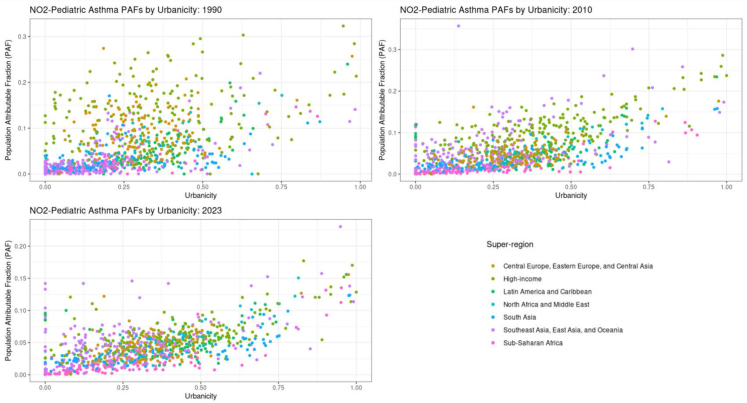
**Urbanicity versus population attributable fraction of asthma YLDs attributable to NO_2_ for individuals <20 years of age for the years 1990, 2010, and 2023. Colors indicate super-regions**.

We estimated that NO_2_-attributable burden was correlated with urbanicity across super-regions (Fig. 3), indicating that NO_2_ remains a notable risk factor for urban populations globally. We observed substantial increases in childhood asthma PAFs with increasing urbanicity. Though this relationship appears to have attenuated over time, NO_2_-attributable burden remains a primarily urban phenomenon (Fig. 3).

**Fig. 4:**
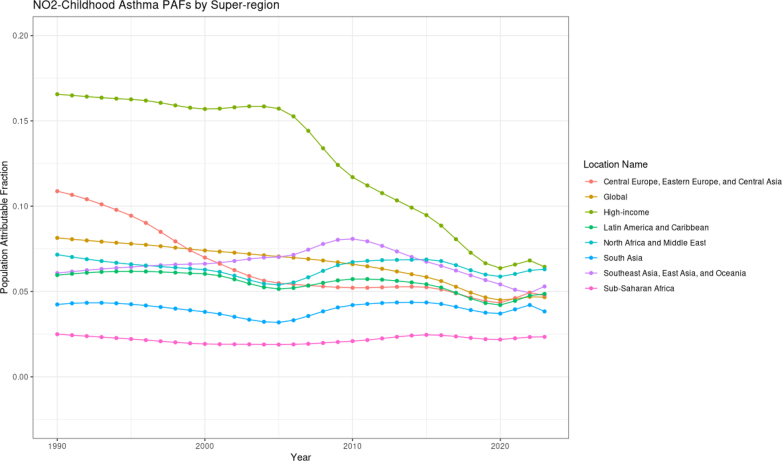
**Population attributable fraction of asthma YLDs attributable to NO_2_ for individuals <20 years of age by super-region from 1990 to 2023**.

Over the last three decades, the number of global NO_2_-attributable asthma YLDs experienced a 30% decline, from 334,000 (95% UI −438,000 to 1,210,000) in 1990 to 233,000 (−250,000 to 956,000) in 2023, and the rate declined from 14.8 (−19.4 to 53.6) per 100,000 to 8.71 (−9.36 to 35.8) YLDs per 100,000 over the same time period. However, the percentage of NO_2_-attributable childhood asthma YLDs as a proportion of all paediatric asthma YLDs exhibited a more moderate global decline, decreasing from 8.14% (−8.24 to 29.5) in 1990 to 4.67% (−3.75 to 20.6) in 2023. Temporal trends diverged widely across super-regions (Fig. 4). NO_2_ PAFs decreased sharply between 1990 and 2023 in the high-income and the central Europe, eastern Europe, and central Asia super-regions, declining from 16.6% (−19.3, 55.7) to 6.45% (−5.69, 28.1) and from 10.9 (−8.99, 42.3) to 4.77 (−3.69, 22.1) in each super-region, respectively. In contrast, PAFs remained mostly stagnant in other super-regions with minor fluctuations over the three decades.

**Fig. 5:**
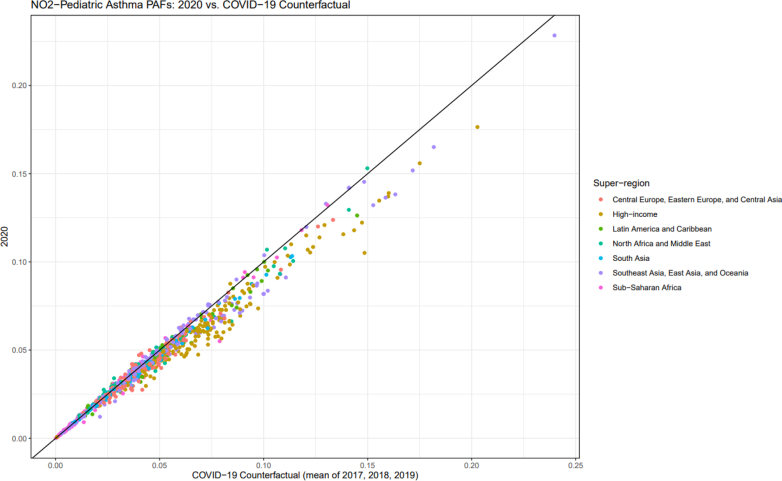
**Comparison of population-attributable fractions of asthma YLDs for individuals <20 years of age estimated for NO_2_ exposure in 2020 and COVID-19 counterfactual levels of NO_2_ exposure (mean of NO_2_ exposure in 2017, 2018, and 2019). Colors denote super-regions**.

We found marked differences between 2020 NO_2_-attributable asthma PAFs for children, as calculated from the data, versus PAFs based on counterfactual NO_2_ exposures chosen to represent likely exposure levels in the absence of the transport and industry changes that occurred during the COVID-19 pandemic (Fig. 5). We particularly observed a clear decrease of about 12% in NO_2_ PAFs estimates from the data in the high-income super-region (Fig. 5). Central Europe, Eastern Europe, and Central Asia as well as South Asia displayed an average decrease of 6% in 2020, while Latin America and the Caribbean and Southeast Asia, East Asia, and Oceania declined by approximately 5%. North Africa and the Middle East decreased by 3% while Sub-Saharan Africa showed a less than 1% decline in PAFs.

**Fig. 6:**
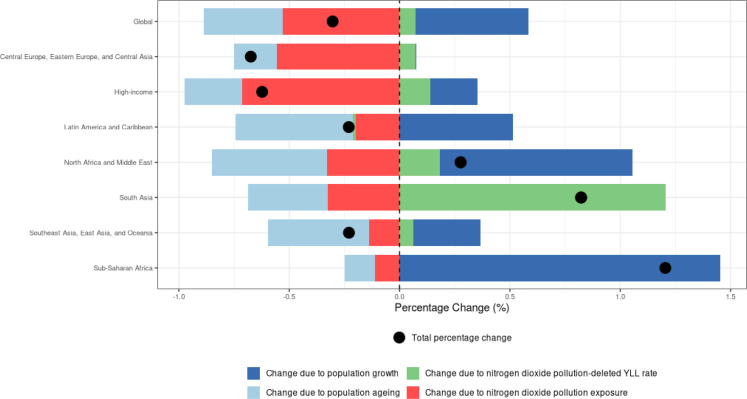
**Drivers of trends in NO_2_-attributable asthma YLDs for individuals under 20 years of age from 1990 to 2023**.

A combination of four factors drove percent change in NO_2_-attributable asthma YLDs for children from 1990 to 2023: population growth, population ageing, NO_2_ pollution-deleted YLD rate (i.e., the change in burden that is not attributable to changes in NO2 exposure, population growth and ageing), and NO_2_ exposure (Fig. 6). Globally, we observed an approximate 30% decrease in NO_2_-attributable paediatric asthma burden, driven by population ageing and reduction in NO_2_ exposure. NO_2_ pollution-deleted YLD, increased in all super regions, except Latin America and Caribbean, highlighting the relevance of other risk factors. For superregions with the largest percent decrease in NO_2_-attributable childhood asthma YLDs during this period (the central Europe, eastern Europe, and central Asia and the high-income super-regions), these changes resulted primarily from reductions in NO_2_ exposure. We also observed reductions in NO_2_-attributable paediatric asthma YLDs in Latin America and the Caribbean and in north Africa and the Middle East due to population ageing, i.e. a lower number of the population below the age of 20 years, and falling rates of asthma YLDs from risk factors other than NO_2_ (and to a lesser extent, NO_2_ pollution reductions). However, these drivers were largely offset by population growth, which attenuated the overall reduction in NO_2_-attributable childhood asthma burden. In south Asia and southeast Asia, east Asia, and Oceania, we observed similar trends; however, for these super-regions, reductions in NO2-attributable burden were less pronounced due to rising NO_2_ exposure levels. In sub-Saharan Africa, population growth was substantial and NO_2_ exposure also increased, leading to a net increase in the NO_2_-attributable childhood asthma burden; other factors were not sufficient to offset the resulting increase in NO2-attributable burden.

## Discussion

This study presents a comprehensive assessment of NO_2_ pollution's impact on risk of paediatric asthma and the resulting NO_2_ pollution-attributable disease burden. We conducted a systematic review of relative risk literature and performed a meta-analysis with improved characterization of between-study heterogeneity. Using these results, we calculated the global burden for 204 countries and territories and analysed the resulting spatiotemporal trends. First, our findings reveal that NO_2_ pollution is a large contributor to YLDs for children globally: relative to other GBD risk factors, NO_2_ was the seventh leading environmental risk factor globally for all-cause YLDs for children and the third leading risk factor globally for childhood asthma YLDs in 2023. Approximately 4.67% of childhood asthma YLDs were attributable to NO_2_ globally in 2023. Second, we found that NO_2_-attributable burden and trends across time varied drastically by super-region. While the high-income and the central Europe, eastern Europe, and central Asia super-regions observed dramatic decreases in NO_2_-attributable asthma YLD since 1990, NO_2_ remains the first and sixth ranked environmental risk factor for children's asthma YLDs in these super-regions, and contributes to 57,100 (95% UI −63,600 to 242,000) and 6570 (−7,050 to 30,200) YLDs for children in 2023 respectively. In contrast, although NO_2_ is only the eighth ranked environmental risk factor in Sub-Saharan Africa, NO_2_-attributable asthma burden is increasing in this super-region, driven by rising NO_2_ exposure and population growth. Third, we estimated that NO_2_-attributable childhood asthma burden is a largely urban phenomenon and that the COVID-19 pandemic resulted in a reduction of NO_2_ PAFs, especially in the high-income super-region due to reductions in motor vehicle traffic.

Estimates presented in this study corroborate spatial patterns in past findings on NO_2_-attributable paediatric asthma burden, although the magnitude of burden estimated here is smaller than reported in previous studies. Two recent burden assessments, Achakulwisut et al.^28^ and Anenberg et al.,^27^ estimated higher attributable burden than this analysis. Achakulwisut and colleagues^28^ estimated a global annual PAF of 13% (95% UI 5.8–16) and 4.0 million (1.8–5.2) NO_2_-attributable incident paediatric asthma cases annually. Anenberg and colleagues^27^ estimated that 8.5% (95% UI 4.3–12.8%) of new pediatric cases were attributable to NO2 in 2019. Our results were lower, with a global PAF of 4.67% (−3.75 to 20.6) and 2.05 million NO_2_ attributable childhood asthma incidence cases in 2023. These considerable differences are due to several factors. First, both previously published studies used Khreis and colleagues’ estimated summary RR of 1.12 (1.05–1.17) per 5 ppb NO_2_ increase, while we estimated a RR of 1.05 (0.99–1.12) per 5 ppb NO_2_ increase. As a unique trait, the Burden of Proof framework incorporates in-between study heterogeneity, i.e. disagreement in study-specific risk-outcome relationships, into uncertainty estimates leading to generally wider uncertainty ranges. Indeed, our study revealed a RR of 1.05 with an uncertainty range from 0.99 to 1.12 when incorporating in-between study heterogeneity and a much narrower uncertainty range from 1.04 to 1.06 when not incorporating in-between study heterogeneity. While we argue that differences in effect magnitude should be reflected in the associated uncertainty, we also caution against comparing risks and uncertainties derived from more traditional estimation methods. Although the lower bound of the uncertainty interval crossing one would typically be interpreted as a null result in conventional analyses, the risk outcome score for the NO_2_–asthma relationship derived in this study provided sufficient evidence to warrant inclusion in the GBD. Furthermore, differences between the pooled risk derived by Khreis et al. and our estimates could result from our analysis including more recent literature and excluded cross-sectional studies, partly explaining differences in the overall risk estimates (27 studies in our analysis versus 17 studies in Khreis and colleagues). In addition, we characterized known variation in input study design characteristics using bias covariates and incorporated remaining unexplained heterogeneity in our final predictions, further leading to differences in pooled RR estimates.

Moreover, previous studies implemented a TMREL of 2 ppb annual average NO_2_ concentration, while this study uses a uniform distribution from 4.6 to 6.2 ppb, contributing to lower estimated PAFs. Finally, substantial differences in NO_2_ exposure datasets and asthma burden predictions result in this study's lower estimated burden. Achakulwisut et al.^28^ used the Larkin et al.^21^ NO_2_ dataset, while both Anenberg et al.^27^ and this study used an updated NO_2_ surface concentration that corrects for overestimated NO_2_ concentrations in rural locations, resulting in lower burden estimates. Additionally, Achakulwisut et al.^28^ produced burden estimates using exposures estimated for 2010–2012, and Anenberg et al. used exposures for 2019 while our study is based on annual exposures from 1990 to 2023. Declining NO_2_ exposure in high-burden super-regions across time likely contributes to lower estimated PAFs and incident cases. Achakulwisut et al.^28^ also used asthma burden estimates from GBD 2015 that are higher than those for GBD 2019 and later iterations, which were used by Anenberg et al. and this study, respectively. Despite these differences, the spatial trends in burden distribution observed in our findings align with those of both Achakulwisut et al.^28^ and Anenberg et al. All studies estimated high burden in the high-income; central Europe, eastern Europe, and central Asia; Latin America and the Caribbean; and north Africa and the Middle East super-regions. Like other available literature, our study also found that NO_2_-attributable childhood asthma burden is most pronounced in urban locations.

This study is global and internally consistent with analyses of other GBD risk factors, strengths that enable comprehensive NO_2_-attributable burden assessment and comparison with other GBD risk factors. This allows us to highlight the prominence of NO_2_ amongst other risk factors as a key contributor to asthma YLDs for children, especially in urban areas. The use of finely resolved NO_2_ exposure estimates also improves the strength of our findings by capturing variation within both urban and rural areas. The variability in effect size between studies is an important contributor to the large uncertainty in estimates of the summary effect size. Our analytical approach, based on the meta-regression tool MR-BRT (Metaregression – Bayesian Regularized Trimmed),^16^^,^^20^ allows us to account for some of this variability by including study-specific bias covariates representing known differences across study-design characteristics. Specifically, we use empirically selected covariates to characterize known variability and then we incorporate the remaining unexplained heterogeneity into our predictions through random effects. This allows a more precise estimation of the pooled effect size and its uncertainty, which is unique to our analysis and constitutes one of the major strengths of this study. Finally, the risk-outcome scoring framework and publication bias detection components of our analysis are particularly useful for standardized comparison of strength of evidence. The framework incorporates both effect size magnitude and uncertainty into a standardized measure of strength of association and the evidence for it, based on the available literature. While our analysis revealed a considerable impact of NO_2_ on paediatric asthma, our findings also indicate the need for further research in this field, particularly across the range of global NO_2_ exposures and across age groups.

Though our meta-analysis of NO_2_-pediatric asthma risk literature is systematic and comprehensive, it remains limited by the availability of NO_2_-asthma relative risk studies. There is a notable scarcity of data for low- and middle-income locations and adult study populations. While our meta regression approach reflects increasing risk with increasing exposure it cannot ultimately confirm causality. Furthermore, our estimation of PAFs relies on several standard assumptions, including the absence of unmeasured confounding, the independence of other risk factors from the exposure, and the plausibility of the counterfactual scenario. While we cannot rule out the possibility of unmeasured confounding, we have adjusted for a comprehensive set of covariates. We also assume that removal of the exposure would not alter the distribution of other risk factors. Importantly, the theoretical minimum risk exposure level (TMREL) used here is based on low exposure levels observed in empirical studies, supporting the realism of this counterfactual scenario.

Due to lack of available evidence, we were unable to account for adult asthma or asthma-attributable mortality in this analysis, meaning that our results likely underestimate the global burden of NO_2_ pollution. Finally, we also need to acknowledge that NO_2_ may be correlated with other exposures as it often co-occurs with a mixture of combustion-related emissions, including particulate matter and other gaseous pollutants potentially leading to confounding in statistical analyses. Hence, while the effects of each individual component cannot be completely disentangled, NO_2_ serves as a robust indicator of traffic- and combustion-related air pollution and results should be interpreted with that in mind.

While the burden of proof assessment does not confirm causality, it provides a robust framework for evaluating the strength of evidence supporting a relationship between NO_2_ exposure and pediatric outcomes. By synthesizing an updated RR estimate that accounts for between-study heterogeneity with granular NO_2_ exposure data, we were able to compare trends in NO_2_-attributable burden across space and time, as well as with burden estimates for other risk factors. We identified a NO_2_ pollution as a global priority amongst environmental risks for children, especially in high-income locations and urban areas. Different from other risk factors, such as household air pollution, ambient particulate matter pollution, or water, sanitation, and hygiene, NO_2_ exposure levels are concentrated in high-income and urban locations instead of developing or rural areas.^23^ Evidence also shows that NO_2_ pollution contributes to urban environmental health disparities, with minority and lower socioeconomic status populations experiencing increased NO_2_ exposure within cities.^29^^,^^30^

With ongoing urbanization and industrialization in many currently low- and middle-income countries, our analysis emphasizes the potential for increasing NO_2_ exposures in low- and middle-income countries. NO_2_ exposure may be especially manageable through reductions in vehicle traffic and increases in public transportation over relatively short timeframes, as indicated by the noticeable reductions in attributable burden in the GBD high-income super region during the COVID-19 pandemic.^31^^,^^32^ In addition, other mitigation measures, such as urban green infrastructure or solid barriers have shown some, albeit small, exposure reduction which can mitigate NO_2._.^33^, ^34^, ^35^ To date, reducing fossil fuel combustion remains the most promising solution to reduce NO_2_ pollution and its associated public health damages.

## Contributors

KB, MB, SW, SA, DG, and PH conceptualized and designed the methodological approach. SW, AP, KB, GHK, and AM conducted the analyses and modelling and had access to and have verified the underlying data. KB, SW, SA, AP, NG, CA, DG, PH, GHK, SAML, AM, and MB collectively contributed to the interpretation of research findings and provided critical feedback on the manuscript. KB and SW led the manuscript writing, with writing contributions to the original draft and review & editing from all co-authors. All authors have read and approved the final manuscript.

## Data sharing statement

To download the data used in these analyses, please visit the Global Health Data Exchange GBD 2023 website at http://ghdx.healthdata.org/. Code used for the analysis can be found here https://ghdx.healthdata.org/gbd-2023/code. Results can be downloaded from https://vizhub.healthdata.org/gbd-results/

## Editor note

The Lancet Group takes a neutral position with respect to territorial claims in published maps and institutional affiliations.

## Declaration of interests

KB has received funding from the Gates Foundation, HEI, NIH and the IHME Innovation fund. MB has received funding from the Gates Foundation, and HEI. SA has received funding from NIH, NASA, NSF, NASA, NOAA, Wellcome Trust, Natural Resources Defense Council, Health Effects Institute. She has also received consulting fees from the US Department of Justice, ICF International and honoraria from the University of Maryland. In addition, she has received payments for her expert testimony from the Department of Justice and travel expenses from the American Geophysical Union. She participates on a Data Safety Monitoring Board or Advisory Board with the Environmental Protection Agency, National Academy of Science, World Health Organization, Clean Air Partners and has leadership or fiduciary role in the American Geophysical Union. DG has received funding from NASA and HEI. GHK has received funding from NASA, HEI and the Wellcome Trust; he has also received consulting fees from the US Department of Justice, The New York State Office of Attorney General, and the California Air Resources Board in addition to payments or honoraria from US Department of State International Visitor Leadership Program.

## References

[1] Khreis H., Kelly C., Tate J., Parslow R., Lucas K., Nieuwenhuijsen M. (2017). Exposure to traffic-related air pollution and risk of development of childhood asthma: a systematic review and meta-analysis. Environ Int.

[2] Anenberg S.C., Henze D.K., Tinney V. (2018). Estimates of the global burden of ambient PM2.5, ozone, and NO_2_ on asthma incidence and emergency room visits. Environ Health Perspect.

[3] Vörös K., Kói T., Magyar D., Rudnai P., Páldy A. (2022). The influence of air pollution on respiratory allergies, asthma and wheeze in childhood in Hungary. Minerva Pediatr.

[4] Deng Q., Lu C., Norbäck D. (2015). Early life exposure to ambient air pollution and childhood asthma in China. Environ Res.

[5] Kravitz-Wirtz N., Teixeira S., Hajat A. (2018). Asthma in the United States, 1990−2014. Int J Environ Res Public Health.

[6] Lavigne E., Gasparrini A., Wang X. (2014). Extreme ambient temperatures and cardiorespiratory emergency room visits: assessing risk by comorbid health conditions in a time series study. Environ Health.

[7] Norbäck D., Lu C., Wang J. (2018). Asthma and rhinitis among Chinese children — indoor and outdoor air pollution and indicators of socioeconomic status (SES). Environ Int.

[8] Mims J.W. (2015). Asthma: definitions and pathophysiology: asthma: definitions and pathophysiology. Int Forum Allergy Rhinol.

[9] Blomberg A., Krishna M.T., Bocchino V. (1997). The inflammatory effects of 2 ppm NO 2 on the airways of healthy subjects. Am J Respir Crit Care Med.

[10] Barck C., Lundahl J., Halldén G., Bylin G. (2005). Brief exposures to NO_2_ augment the allergic inflammation in asthmatics. Environ Res.

[11] Ezratty V., Guillossou G., Neukirch C. (2014). Repeated nitrogen dioxide exposures and eosinophilic airway inflammation in asthmatics: a randomized crossover study. Environ Health Perspect.

[12] Strand V., Rak S., Svartengren M., Bylin G. (1997). Nitrogen dioxide exposure enhances asthmatic reaction to inhaled allergen in subjects with asthma. Am J Respir Crit Care Med.

[13] Schwartz J. (2004). Air pollution and children's health. Pediatrics.

[14] Boogaard H., Patton A.P., Atkinson R.W. (2022). Long-term exposure to traffic-related air pollution and selected health outcomes: a systematic review and meta-analysis. Environ Int.

[15] Ferrari A.J., Santomauro D.F., Aali A. (2024). Global incidence, prevalence, years lived with disability (YLDs), disability-adjusted life-years (DALYs), and healthy life expectancy (HALE) for 371 diseases and injuries in 204 countries and territories and 811 subnational locations, 1990–2021: a systematic analysis for the global burden of disease study 2021. Lancet.

[16] Zheng P., Afshin A., Biryukov S. (2022). The burden of proof studies: assessing the evidence of risk. Nat Med.

[17] Brauer M., Roth G.A., Aravkin A.Y. (2024). Global burden and strength of evidence for 88 risk factors in 204 countries and 811 subnational locations, 1990–2021: a systematic analysis for the global burden of disease study 2021. Lancet.

[18] Murray C.J., Ezzati M., Lopez A.D., Rodgers A., Vander Hoorn S. (2003). Comparative quantification of health risks: conceptual framework and methodological issues. Popul Health Metr.

[19] Page M.J., McKenzie J.E., Bossuyt P.M. (2021). The PRISMA 2020 statement: an updated guideline for reporting systematic reviews. BMJ.

[20] Zheng P., Barber R., Sorensen R.J.D. (2021). Trimmed Constrained Mixed Effects Models: Formulations and Algorithms. Comput graph stat.

[21] Larkin A., Geddes J.A., Martin R.V. (2017). A global land use regression model for nitrogen dioxide air pollution. Environ Sci Technol.

[22] Larkin A., Anenberg S., Goldberg D.L., Mohegh A., Brauer M., Hystad P. (2023). A global spatial-temporal land use regression model for nitrogen dioxide air pollution. Front Environ Sci.

[23] GBD 2023 Disease and Injury and Risk Factor Collaborators (2023). Global incidence, prevalence, non-fatal burden and health-adjusted life expectancy for 376 diseases and injuries, including risk-attributable burden for 88 risk factors in 204 countries and territories, and 660 subnational locations, 1990–2023: a systematic analysis for the global burden of disease study. Lancet.

[24] Mohegh A., Goldberg D., Achakulwisut P., Anenberg S.C. (2020). Sensitivity of estimated NO_2_-attributable pediatric asthma incidence to grid resolution and urbanicity. Environ Res Lett.

[25] Geography and Environmental Science (2018).

[26] Egger M., Davey Smith G., Schneider M., Minder C. (1997). Bias in meta-analysis detected by a simple, graphical test. BMJ.

[27] Anenberg S.C., Mohegh A., Goldberg D.L. (2022). Long-term trends in urban NO_2_ concentrations and associated paediatric asthma incidence: estimates from global datasets. Lancet Planet Health.

[28] Achakulwisut P., Brauer M., Hystad P., Anenberg S.C. (2019). Global, national, and urban burdens of paediatric asthma incidence attributable to ambient NO_2_ pollution: estimates from global datasets. Lancet Planet Health.

[29] Kerr G.H., van Donkelaar A., Martin R.V. (2024). Increasing racial and ethnic disparities in ambient air pollution-attributable morbidity and mortality in the United States. Environ Health Perspect.

[30] Hajat A., Hsia C., O'Neill M.S. (2015). Socioeconomic disparities and air pollution exposure: a global review. Curr Environ Health Rep.

[31] Bassani C., Vichi F., Esposito G., Montagnoli M., Giusto M., Ianniello A. (2021). Nitrogen dioxide reductions from satellite and surface observations during COVID-19 mitigation in Rome (Italy). Environ Sci Pollut Res.

[32] Burns J., Hoffmann S., Kurz C., Laxy M., Polus S., Rehfuess E. (1994). COVID-19 mitigation measures and nitrogen dioxide - a quasi-experimental study of air quality in Munich, Germany. Atmos Environ.

[33] Jeanjean A.P.R., Gallagher J., Monks P.S., Leigh R.J. (2017). Ranking current and prospective NO_2_ pollution mitigation strategies: an environmental and economic modelling investigation in Oxford Street, London. Environ Pollut.

[34] Gong C., Xian C., Ouyang Z. (2022). Assessment of NO_2_ purification by urban forests based on the i-Tree eco model: case study in Beijing, China. Forests.

[35] Rao M., George L.A., Shandas V., Rosenstiel T.N. (2017). Assessing the potential of land use modification to mitigate ambient NO_2_ and its consequences for respiratory health. Int J Environ Res Public Health.

[36] The George Washington University High Performance Computing, Cluster (2020). https://Arxiv.Org/Abs/2003.13629/.

