# Supplementary material for: Global, regional and national estimates of the burden of childhood asthma attributable to NO2 exposure for 204 countries and territories from 1990 to 2023: a Global Burden of Disease study 2023
Source: eClinicalMedicine. 2025 Nov 1;90:103580. doi: 10.1016/j.eclinm.2025.103580 (PMC12617645; doi:10.1016/j.eclinm.2025.103580)
Supplement: Multimedia component 1 [file mmc1.docx]

**Global, regional and national estimates of the burden of childhood asthma attributable to NO2 exposure for 204 countries and territories from 1990 to 2023:**
**a Global Burden of Disease 2023 study**

*Katrin Burkart^1^, Sarah Wozniak^1^, Susan Anenberg^2^, Ana Pereda^1^, Nora Gilbertson^1^, Charlie Ashbaugh^1^, Daniel Goldberg^2^, Perry Hystad^3^, Gaige Kerr^2^, Susan McLaughlin^1^, Arash Mohegh^2^, Michael Brauer^1,4^*

1 Institute for Health Metrics and Evaluation, University of Washington, Seattle, WA, USA

2  Milken Institute School of Public Health, Department of Environmental and Occupational Health, George Washington University, Washington DC, USA

3 College of Health, Oregon State University, Corvallis, OR , USA

4 School of Population and Public Health, The University of British Columbia, Vancouver BC, Canada

**Systematic Review**

***Data Extraction***

As outlined in the main manuscript, we first extracted the 31 NO_2_-related component studies from a recent (2017) and comprehensive meta-analysis of traffic-related air pollution’s effects on childhood asthma development conducted by Khreis and colleagues.^1^ We then searched PubMed and Embase for updates to relevant literature published from September 9, 2016, to December 19, 2019, using the search string below.

Search string: (((((("child*") AND "air pollution") AND "asthma")) OR ((("child*") AND "air quality") AND "asthma")) OR ((("child*") AND "vehicle emissions") AND "asthma")) OR ((("child*") AND "ultra-fine particles") AND "asthma")  .

We excluded all cross-sectional studies, as this type of study design is less robust. Apart from this exclusion, we employed inclusion and exclusion criteria identical to those used in the Khreis et al., ^1^ meta-analysis. Input data included in the GBD 2021 NO_2_-childhood asthma relative risk analysis are as follows, with all sources detailed below:

We extracted risk estimates related to the incidence or prevalence of childhood asthma given preceding exposure to ambient NO2 pollution. These included estimates of odds ratios (ORs), hazard ratios (HRs), or relative risks (RRs). For the purpose of our analysis, we assumed these measures were equivalent. We also extracted study-specific NO2 exposure distributions and used these to scale each estimated effect size to inform the combined RR curve between the 5^th^ and 95^th^ percentiles of its study’s NO2 distribution.

Additionally, we extracted a set of study-specific covariates for use in characterizing unexplained between-study heterogeneity. Each of these was coded as a binary indicator variable for empirical covariate selection using Lasso penalties. The full set of covariates is detailed below. In addition to the ten covariates widely use across GBD risk factor teams and used for the other air pollution risk factors within the GBD (subpopulation, exposure population, self-reported exposure, exposure time, self-reported outcome, outcome unblinded, randomized study design, uncontrolled confounders, selection bias, and follow-up duration), we extracted an additional covariate specifically pertinent to NO2 as a risk factor for pediatric asthma (whether or not the study controlled for particulate matter exposure, PM2.5 or PM10).


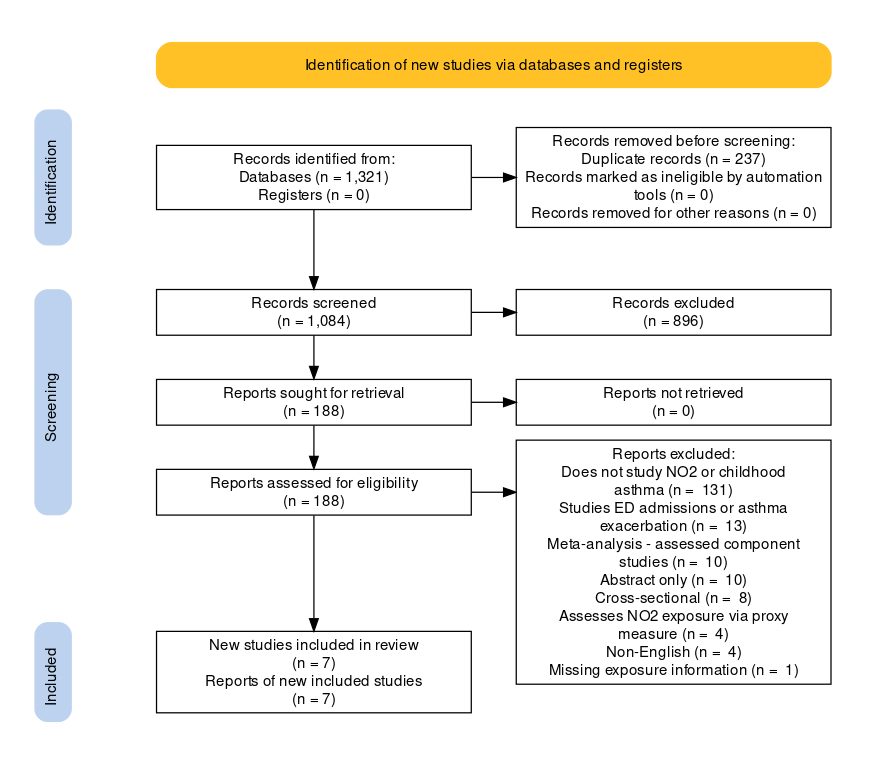


***Figure S1. PRISMA diagram for* NO_2_*-pediatric asthma systematic review***

***Table S1. Study-specific heterogeneity covariates extracted.***

| **Covariate Extracted** | **Definition** |
| --- | --- |
| Subpopulation | 0: general population  1: subgroup (e.g. high risk group) |
| Exposure population | 0: study uses individual exposure (</= 500m radius or postal code)  1: study uses population-level exposure |
| Exposure time | 0: exposure measured multiple times throughout study  1: exposure measured only at baseline |
| Self-reported outcome | 0: outcome based on death certificate or medical record  1: self-reported outcome |
| Prevalence | 0: prevalence was assessed  1: incidence was assessed |
| Randomized study design | 0: randomized study  1: non-randomized study |
| Confounding controlled | 0: controlled for age, sex, education, income (SES), and other critical determinants (SHS, parental/maternal asthma/allergy)  1: controlled for age, sex, education, income (SES), and other critical determinants (SHS, parental/maternal asthma/allergy)  2: controlled for only age and sex |
| Loss to follow-up | 0: follow up >= 95%  1: 85% - 95% follow-up  2: less than 85% follow-up |
| Follow-up duration | 0: greater or equal to than 5 years of cohort follow-up  1: less than 5 years of cohort follow-up |
| PM controlled | 0: controlled for particulate matter exposure (any size PM)  1: not controlled for PM |
| Exposure postnatal | 0 for exposure measured during postnatal period; 1 for exposure measured during prenatal period |
| RR | 0: if outcome measure was RR  1: all other measures |
| OR | 0: if outcome measure was OR  1: all other measures |
| HR | 0: if outcome measure was HR  1: all other measures |

***Results***

The results of our systematic review included prospective and retrospective cohort studies examining the relationship between ambient NO2 pollution and childhood asthma incidence or prevalence. XX additional sources in XX locations were included beyond those identified in Khreis et al. 2017, for a total of 35 studies across 20 locations spanning the years 1987-2019. The full list of studies is available in the appendix.

***Table 2. Studies included in NO2-pediatric asthma meta-analysis.***

| *Study number* | *Reference* |
| --- | --- |
| *1* | *Gehring U, Wijga AH, Hoek G, Bellander T, Berdel D, Brüske I, Fuertes E, Gruzieva O, Heinrich J, Hoffmann B, de Jongste JC, Klümper C, Koppelman GH, Korek M, Krämer U, Maier D, Melén E, Pershagen G, Postma DS, Standl M, von Berg A, Anto JM, Bousquet J, Keil T, Smit HA, Brunekreef B. Exposure to air pollution and development of asthma and rhinoconjunctivitis throughout childhood and adolescence: a population-based birth cohort study. Lancet Respir Med. 2015; 3(12): 933-42.* |
| *2* | *Carlsten C, Dybuncio A, Becker A, Chan-Yeung M, Brauer M. Traffic-related air pollution and incident asthma in a high-risk birth cohort. Occup Environ Med. 2011; 68(4): 291-5.* |
| *3* | *Norbäck D, Lu C, Wang J, Zhang Y, Li B, Zhao Z, Huang C, Zhang X, Qian H, Sun Y, Sundell J, Deng Q. Asthma and rhinitis among Chinese children - Indoor and outdoor air pollution and indicators of socioeconomic status (SES). Environ Int. 2018; 115: 1-8.* |
| *4* | *Deng Q, Lu C, Ou C, Chen L, Yuan H. Preconceptional, prenatal and postnatal exposure to outdoor and indoor environmental factors on allergic diseases/symptoms in preschool children. Chemosphere. 2016; 152: 459-67.* |
| *5* | *Liu W, Huang C, Hu Y, Fu Q, Zou Z, Sun C, Shen L, Wang X, Cai J, Pan J, Huang Y, Chang J, Sun Y, Sundell J. Associations of gestational and early life exposures to ambient air pollution with childhood respiratory diseases in Shanghai, China: A retrospective cohort study. Environ Int. 2016; 92-93: 284-93.* |
| *6* | *Clark NA, Demers PA, Karr CJ, Koehoorn M, Lencar C, Tamburic L, Brauer M. Effect of Early Life Exposure to Air Pollution on Development of Childhood Asthma. Environ Health Perspect. 2010; 118(2): 284-90.* |
| *7* | *Deng Q, Lu C, Norbeck D, Bornehag CG, Zhang Y, Liu W, Yuan H, Sundell J. Early life exposure to ambient air pollution and childhood asthma in China. Environ Res. 2015; 143: 83-92.* |
| *8* | *Nishimura KK, Galanter JM, Roth LA, Oh SS, Thakur N, Nguyen EA, Thyne S, Farber HJ, Serebrisky D, Kumar R, Brigino-Buenaventura E, Davis A, LeNoir MA, Meade K, Rodriguez-Cintron W, Avila PC, Borrell LN, Bibbins-Domingo K, Rodriguez-Santana JR, Sen Åš, Lurmann F, Balmes JR, Burchard EG. Early-life air pollution and asthma risk in minority children. The GALA II and SAGE II studies. Am J Respir Crit Care Med. 2013; 188(3); 309-18.* |
| *9* | *Ranzi A, Porta D, Badaloni C, Cesaroni G, Lauriola P, Davoli M, Forastiere F. Exposure to air pollution and respiratory symptoms during the first 7 years of life in an Italian birth cohort. Occup Environ Med. 2014; 71(6): 430-6.* |
| *10* | *Morgenstern V, Zutavern A, Cyrys J, Brockow I, Koletzko S, KrÃ¤mer U, Behrendt H, Herbarth O, von Berg A, Bauer CP, Wichmann HE, Heinrich J, GINI Study Group, LISA Study Group. Atopic diseases, allergic sensitization, and exposure to traffic-related air pollution in children.Â Am J Respir Crit Care Med. 2008; 177(12): 1331-7.* |
| *11* | *Krämer U, Sugiri D, Ranft U, Krutmann J, von Berg A, Berdel D, Behrendt H, Kuhlbusch T, Hochadel M, Wichmann HE, Heinrich J, GINIplus and LISAplus study groups. Eczema, respiratory allergies, and traffic-related air pollution in birth cohorts from small-town areas. J Dermatol Sci. 2009; 56(2): 99-105.* |
| *12* | *Lavigne É, Bélair MA, Rodriguez Duque D, Do MT, Stieb DM, Hystad P, van Donkelaar A, Martin RV, Crouse DL, Crighton E, Chen H, Burnett RT, Weichenthal S, Villeneuve PJ, To T, Brook JR, Johnson M, Cakmak S, Yasseen AS 3rd, Walker M. Effect modification of perinatal exposure to air pollution and childhood asthma incidence. Eur Respir J. 2018.* |
| *13* | *Fuertes E, Standl M, Cyrys J, Berdel D, von Berg A, Bauer CP, KrÃ¤mer U, Sugiri D, Lehmann I, Koletzko S, Carlsten C, Brauer M, Heinrich J. A longitudinal analysis of associations between traffic-related air pollution with asthma, allergies and sensitization in the GINIplus and LISAplus birth cohorts. PeerJ. 2003; 1: e193.* |
| *14* | *Mölter A, Agius R, de Vocht F, Lindley S, Gerrard W, Custovic A, Simpson A. Effects of long-term exposure to PM10 and NO2 on asthma and wheeze in a prospective birth cohort. J Epidemiol Community Health. 2014; 68(1): 21-8.* |
| *15* | *Clougherty JE, Levy JI, Kubzansky LD, Ryan PB, Suglia SF, Canner MJ, Wright RJ. Synergistic effects of traffic-related air pollution and exposure to violence on urban asthma etiology.Â Environ Health Perspect. 2007; 115: 1140-6.* |
| *16* | *Oftedal B, Nystad W, Brunekreef B, Nafstad P. Long-term traffic-related exposures and asthma onset in schoolchildren in Oslo, Norway. Environ Health Perspect. 2009; 117(5): 839-44.* |
| *17* | *Kravitz-Wirtz N, Teixeira S, Hajat A, Woo B, Crowder K, Takeuchi D. Early-Life Air Pollution Exposure, Neighborhood Poverty, and Childhood Asthma in the United States, 19902014.Â Int J Environ Res Public Health. 2018; 15(6).* |
| *18* | *Brauer M, Hoek G, Smit HA, de Jongste JC, Gerritsen J, Postma DS, Kerkhof M, Brunekreef B. Air pollution and development of asthma, allergy and infections in a birth cohort. Eur Respir J. 2007; 29(5): 879-88.* |
| *19* | *Sbihi H, Koehoorn M, Tamburic L, Brauer M. Asthma Trajectories in a Population-based Birth Cohort. Impacts of Air Pollution and Greenness.Â Am J Respir Crit Care Med. 2017; 195(5): 607-613.* |
| *20* | *Shima M, Adachi M. Effect of outdoor and indoor nitrogen dioxide on respiratory symptoms in schoolchildren. Int J Epidemiol. 2000; 29(5): 862-70.* |
| *21* | *Shima M, Nitta Y, Ando M, Adachi M. Effects of air pollution on the prevalence and incidence of asthma in children. Arch Environ Health. 2002; 57(6): 529-35.* |
| *22* | *Jerrett M, Shankardass K, Berhane K, Gauderman WJ, Kunzli N, Avol E, Gilliland F, Lurmann F, Molitor JN, Molitor JT, Thomas DC, Peters J, McConnell R. Traffic-Related Air Pollution and Asthma Onset in Children: A Prospective Cohort Study with Individual Exposure Measurement. Environ Health Perspect. 2008; 116(10): 1433-8.* |
| *23* | *McConnell R, Islam T, Shankardass K, Jerrett M, Lurmann F, Gilliland F, Gauderman J, Avol E, KÃ¼nzli N, Yao L, Peters J, Berhane K. Childhood incident asthma and traffic-related air pollution at home and school. Environ Health Perspect. 2010; 118(7): 1021-6.* |
| *24* | *Tétreault LF, Doucet M, Gamache P, Fournier M, Brand A, Kosatsky T, Smargiassi A. Childhood Exposure to Ambient Air Pollutants and the Onset of Asthma: An Administrative Cohort Study in Québec. Environ Health Perspect. 2016; 124(8): 1276-82.* |
| *25* | *To T, Zhu J, Stieb D, Gray N, Fong I, Pinault L, Jerrett M, Robichaud A, MÃ©nard R, van Donkelaar A, Martin RV, Hystad P, Brook JR, Dell S. Early Life Exposure to Air Pollution and Incidence of Childhood Asthma, Allergic Rhinitis and Eczema.Â Eur Respir J. 2019.* |
| *26* | *Dell SD, Jerrett M, Beckerman B, Brook JR, Foty RG, Gilbert NL, Marshall L, Miller JD, To T, Walter SD, Stieb DM. Presence of other allergic disease modifies the effect of early childhood traffic-related air pollution exposure on asthma prevalence. Environ Int. 2014; 65: 83-92.* |
| *27* | *Voros K, Koi T, Magyar D, Rudnai P, Paldy A. The influence of air pollution on respiratory allergies, asthma and wheeze in childhood in Hungary.Â Minerva Pediatr. 2019.* |

We made two adjustments to prevent a single cohort or study from unduly weighting the final estimate. When multiple individual publications were available for the same cohort, we included only the maximum RR estimate across follow-up time to account for patterns of asthma incidence and remission across age. Additionally, the standard errors of observations from studies with multiple observations for a single cohort reporting an unstratified sample size were weighted by the inverse square root of *n*, where *n* is the total number of observations for a given cohort.

We performed covariate selection to empirically identify significant covariates from those extracted to quantify between-study heterogeneity. The MR-BRT automated covariate selection tool implements a two-step process. First, a series of loosening Lasso penalty parameters are applied to a log-linear meta-regression on all input effect size observations to rank covariates. Once the covariates are ranked, a stepwise selection is performed, where covariates are brought in one at a time. Covariates with a non-zero coefficient are tested for significance using a Gaussian prior (significance threshold = 0.05). A Gaussian prior was also used on each covariate’s coefficient during curve fitting (mean = 0, variance = 0.1 multiplied by the standard deviation of the beta from the initial log-linear meta-regression) The process is terminated once any bias covariate fails to be statistically significant

The within-study sampling errors are assumed to be normally distributed, i.e., the reported standard errors of the RRs $\in_{i,j}\sim N\left( 0,{\sigma_{i,j}^{2}} \right)$ We also assume the random effects $\upsilon_{i}$ are approximately normally distributed as $\upsilon_{i} \sim N(0,\gamma)$ ui N(0, ), wth gamma an unknown shared variance. In the linear mixed-effects model used for the metaregression, there is no link function. Relative risks are transformed to log space and Bayesian regularized splines are used to obtain the shape of the exposure versus log relative risk relationship, whether the shape is linear or nonlinear.

$Type equation here.$We generated 1000 final predictions of the effect size at 5 ppb NO_2_ exposure for use in calculating burden estimates. To better characterize uncertainty in model predictions, these draws were created incorporating predictions of between-study heterogeneity and Fisher information.

Briefly, we used a combination of Lasso regression and Gaussian priors to empirically identify significant covariates from those extracted (Table SX) to quantify heterogeneity.

The Larkin dataset has been previously shown to overestimate NO_2_ concentrations in rural areas,^15^ so to account for this, satellite- and chemical transport-based products were used to generate rural NO_2_ surface concentration estimates. These rural estimates were scaled to full seasonal and annual coverage using satellite products (for details, see appendix) before combination with the Larkin dataset.^14,17^

***Table S3: Study characteristics of studies included in Burden of Proof assessment***

| **Study** | **Study type** | **Location** | **Measure** | **Sample size** | **NO_2_ concentration (95% CI)** | **Sub-**  **population** | **Exposure population** | **PM controlled** | **Self-reported exposure** | **Confounding controlled** | **Selection bias** | **Exposure postnatal** | **Follow-up duration** |
| --- | --- | --- | --- | --- | --- | --- | --- | --- | --- | --- | --- | --- | --- |
| Gehring  et al. 2015 | prospective cohort | Sweden | OR | 4,010 | 21.2  (14.8 - 27.7) | 0 | 0 | 1 | 1 | 0 | 0 | 0 | 0 |
| Carlsten  et al. 2011 | prospective cohort, intervention study | Canada | OR | 84 | 32.6  (27.4 - 37.8) | 1 | 0 | 1 | 0 | 2 | 2 | 0 | 0 |
| Norbäck et al. 2018 | retrospective cohort | China | OR | 39,782 | 47.0  (23.8 - 68.0) | 0 | 1 | 1 | 1 | 1 | 2 | 0 | 1 |
| Deng et al. 2016 | retrospective cohort | Hunan | OR | 2,598 | 43.0  (36.9 - 49.1) | 0 | 0 | 1 | 1 | 1 | 2 | 1 | 1 |
| Liu et al. 2016 | retrospective cohort | Shanghai | OR | 2,527 | 56.0  (46.8 - 65.2) | 0 | 0 | 0 | 1 | 1 | 2 | 1 | 0 |
| Clark et al. 2010 | prospective cohort | Canada | OR | 16,806 | 31.7  (24.2 - 39.2) | 0 | 0 | 1 | 0 | 0 | 2 | 1 | 1 |
| Deng et al. 2015 | retrospective cohort | Hunan | OR | 1,561 | 46.0  (39.0 - 53.0) | 0 | 0 | 1 | 1 | 2 | 2 | 1 | 1 |
| Nishimura et al. 2013 | retrospective cohort | Puerto Rico | OR | 1,336 | 9.9  (0.0 - 22.8) | 1 | 0 | 1 | 1 | 1 | 2 | 0 | 0 |
| Ranzi et al. 2014 | prospective cohort | Italy | OR | 486 | 44.6  (35.0 - 54.2) | 0 | 0 | 1 | 1 | 1 | 2 | 0 | 0 |
| Morgenstern et al. 2008 | prospective cohort | Germany | OR | 2,436 | 34.6  (27.7 - 41.5) | 0 | 0 | 1 | 1 | 1 | 2 | 0 | 0 |
| Krämer et al. 2009 | prospective cohort | Germany | RR | 2,059 | 24.0  (17.1 - 30.9) | 0 | 0 | 1 | 1 | 1 | 2 | 0 | 0 |
| Lavigne et al. 2018 | retrospective cohort | Canada | HR | 761,172 | 13.2  (0.4 - 26.0) | 0 | 0 | 1 | 0 | 2 | 0 | 1 | 0 |
| Fuertes et al. 2003 | prospective cohort | Germany | OR | 423 | 20.8  (13.9 - 27.7) | 0 | 0 | 1 | 1 | 1 | 2 | 0 | 0 |
| Mölter et al. 2014 | prospective cohort | North West England | OR | 927 | 21.7  (20.4 - 23.0) | 0 | 0 | 1 | 1 | 1 | 2 | 0 | 0 |
| Clougherty et al. 2007 | prospective cohort | Massachusetts | OR | 413 | 27.5  (17.3 - 37.7) | 1 | 0 | 1 | 1 | 2 | 2 | 0 | 0 |
| Oftedal et al. 2009 | prospective cohort | Oslo | RR | 2,871 | 39.3  (32.4 - 46.2) | 0 | 0 | 1 | 1 | 0 | 2 | 0 | 0 |
| Kravitz-Wirtz et al. 2018 | retrospective cohort | United States of America | OR | 4,535 | 21.9  (11.7 - 32.1) | 0 | 0 | 1 | 1 | 1 | 2 | 0 | 0 |
| Brauer et al. 2017 | prospective cohort | Netherlands | OR | 2,575 | 25.2  (18.3 - 32.1) | 0 | 0 | 1 | 1 | 1 | 2 | 0 | 1 |
| Sbihi H et al. 2017 | prospective cohort | Canada | OR | 68,195 | 33.2  (23.0 - 43.4) | 0 | 0 | 1 | 0 | 2 | 1 | 1 | 0 |
| Shima et al. 2000 | prospective cohort | Chiba | OR | 434 | 21.2  (11.3 - 31.1) | 0 | 1 | 1 | 1 | 1 | 2 | 0 | 1 |
| Shima et al. 2002 | prospective cohort | Chiba | OR | 1,539 | 21.2  (11.3 - 31.1) | 0 | 1 | 1 | 1 | 2 | 2 | 0 | 1 |
| Jerrett et al. 2008 | prospective cohort | California | HR | 196 | 29.9  (22.7 - 37.1) | 0 | 0 | 1 | 1 | 0 | 1 | 0 | 0 |
| McConnell et al. 2010 | prospective cohort | California | HR | 2,497 | 20.4  (8.7 - 32.3) | 0 | 0 | 1 | 1 | 0 | 2 | 0 | 1 |
| Tétreault et al.  2016 | prospective cohort | Canada | HR | 1,286 | 26.5  (19.0 - 34.1) | 0 | 0 | 1 | 0 | 0 | 2 | 0 | 0 |
| To et al. 2019 | prospective cohort | Canada | HR | 216,746 | 15.5  (4.5 - 28.4) | 0 | 0 | 1 | 0 | 2 | 2 | 0 | 0 |
| Dell et al. 2014 | retrospective cohort | Canada | OR | 1,479 | 18.3  (13.9 - 22.7) | 0 | 0 | 1 | 1 | 0 | 1 | 0 | 0 |
| Voros et al. 2019 | retrospective cohort | Hungary | OR | 6,737 | 25.9  (19.0 - 32.8) | 0 | 1 | 1 | 1 | 0 | 0 | 0 | 0 |

***Risk outcome scoring***


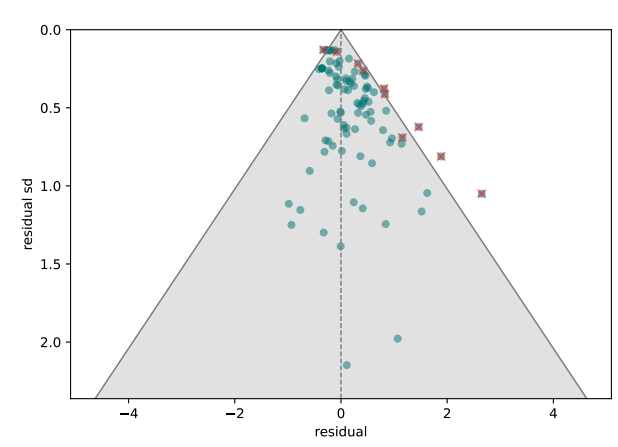


**Figure S2. NO_2_ pollution and childhood asthma risk literature funnel plot. Gray points indicate input data observations. Red Xs denote the 10% of observations trimmed during model fitting.**


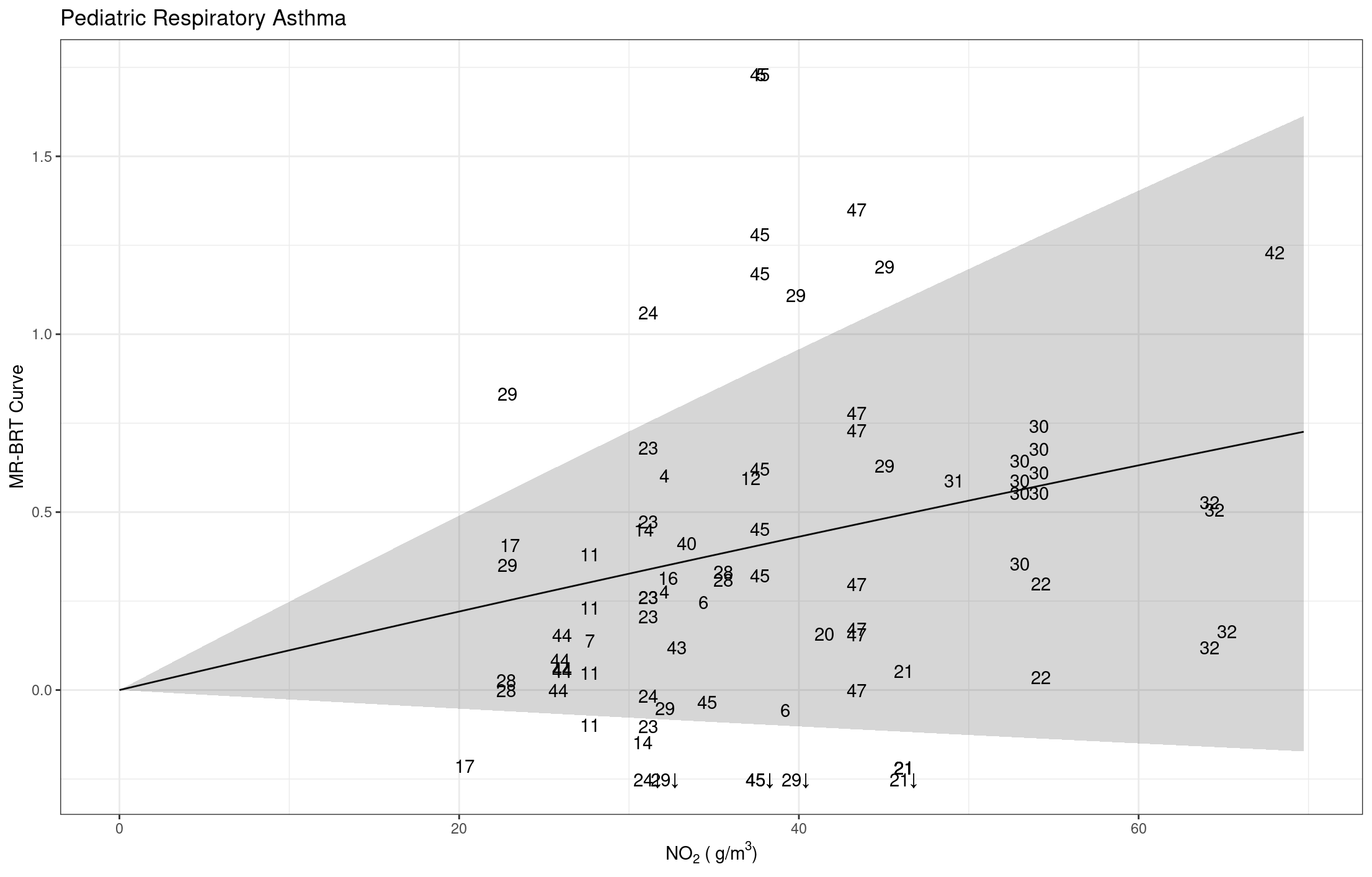


**Figure S3: Nitrogen dioxide pollution and childhood asthma log-linear relative risk curve**

Each number represents one study effect size. Each study’s effect size is plotted at the 95^th^ percentile of its study-specific NO_2_ exposure distribution. The relative risk is plotted relative to the predicted relative risk at the 5^th^ percentile of the study-specific exposure distribution.

***Theoretical Minimum Risk Exposure Level***

To calculate population attributable fractions (PAFs), we used a theoretical minimum risk exposure level (TMREL) of a uniform distribution between 4.6 - 6.2 ppb. This TMREL was calculated by imputing the 0.1 and 5^th^ percentiles of study-specific exposure distributions (when the minimum or 5^th^ percentile was unreported) for all NO_2_ cohort studies that reported a median and standard deviation. The TMREL bounds are the means of the minimum/0.1 and 5^th^ percentiles for the five studies with the lowest reported values. This TMREL calculation method is consistent with that used for the other GBD ambient air pollution risk factors (ambient particulate matter pollution and ozone).^2^

When calculating an evidence score, we observed significant association between observation residuals and their standard errors, indicating the presence of publication bias (p-value = 0.014, Egger mean = -0.244, Egger SD = 0.111). We adjusted for this bias by filling an additional point before refitting the model with the adjusted dataset. This adjusted model was used only to generate an evidence score, not to calculate population attributable fractions. The final evidence score is -0.397, which corresponds to a star rating of 1.


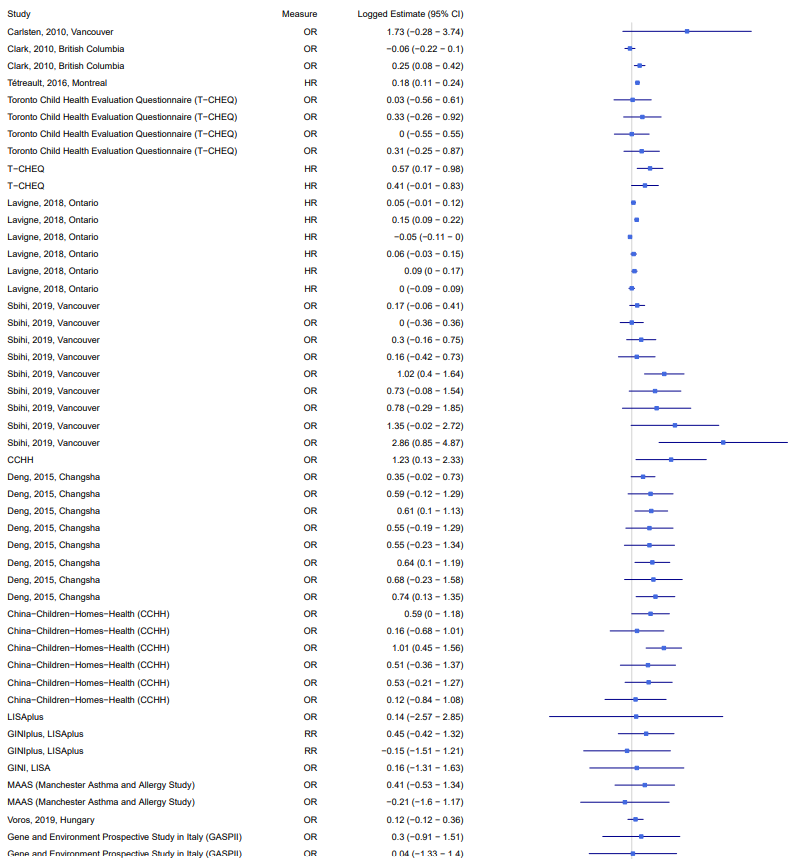


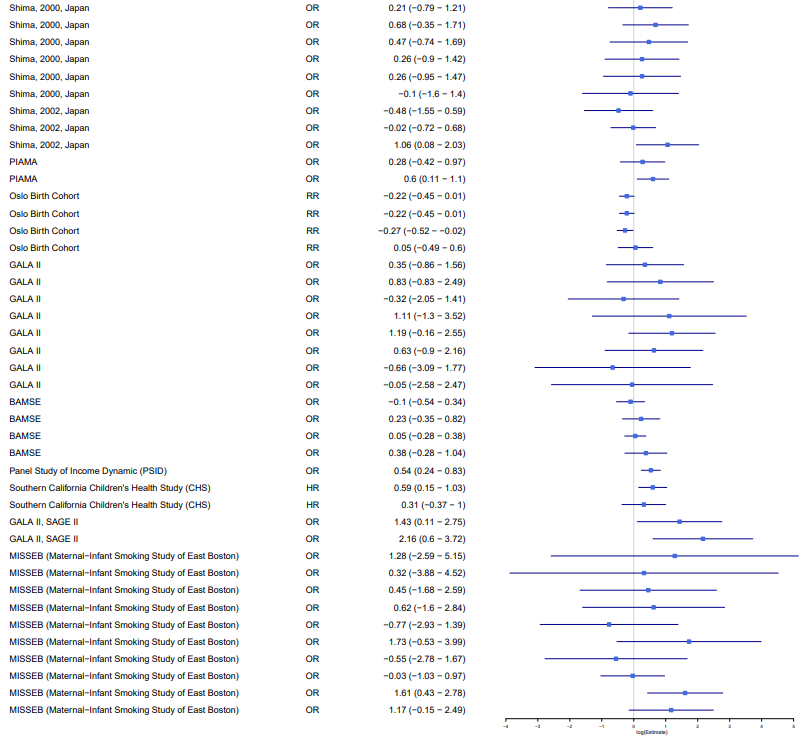


**Figure S4: Forest plot displaying risks estimates from studies included in the burden of proof assessment. The pooled RR across all studies is 1.09 (95%CI: 1.07-1.11)**


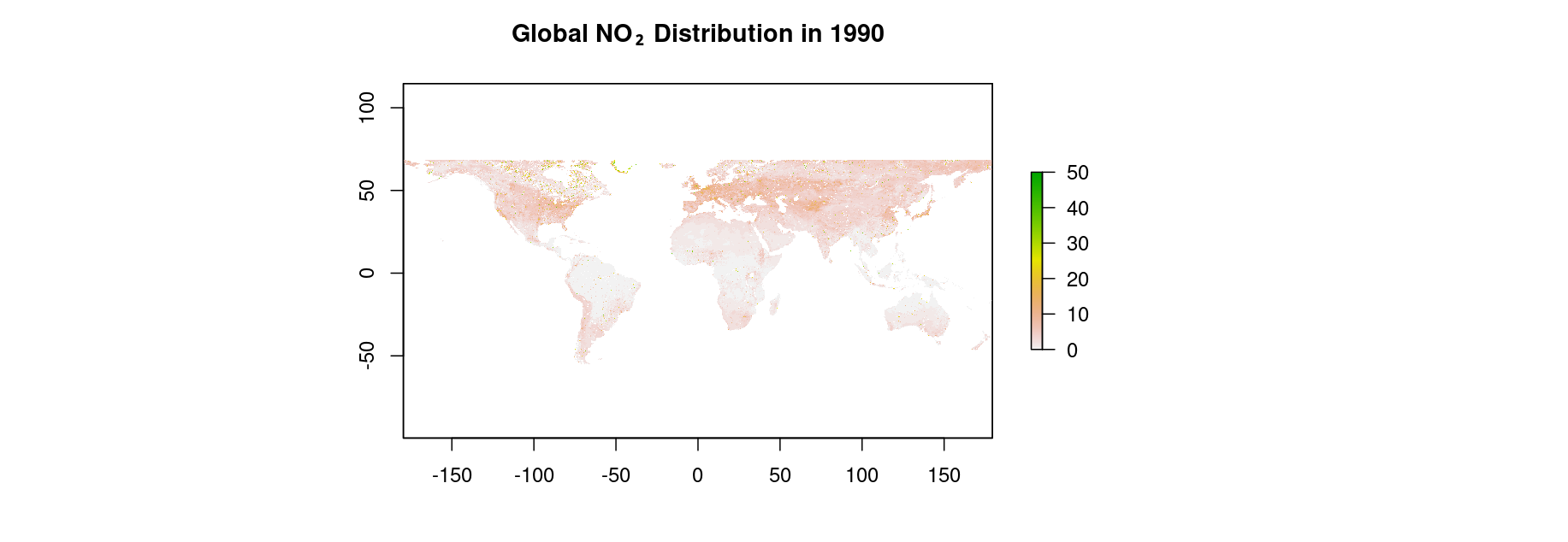


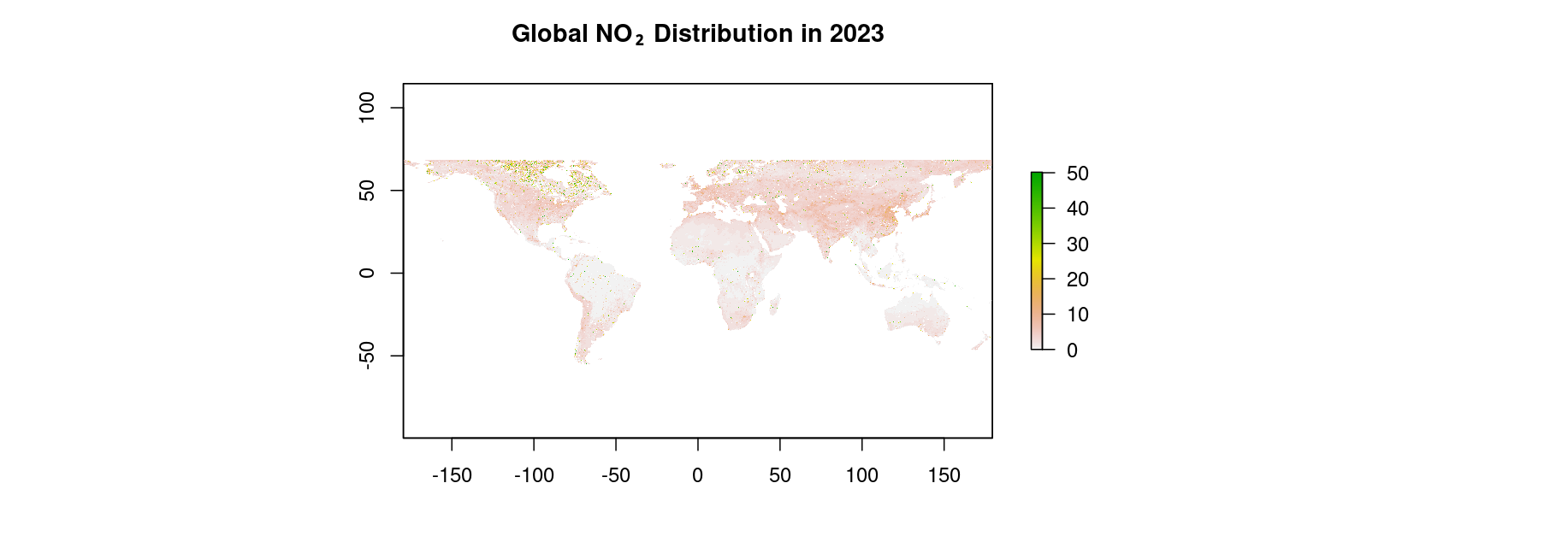


**Figure S5. NO2-exposure maps for 1990 and 2023**


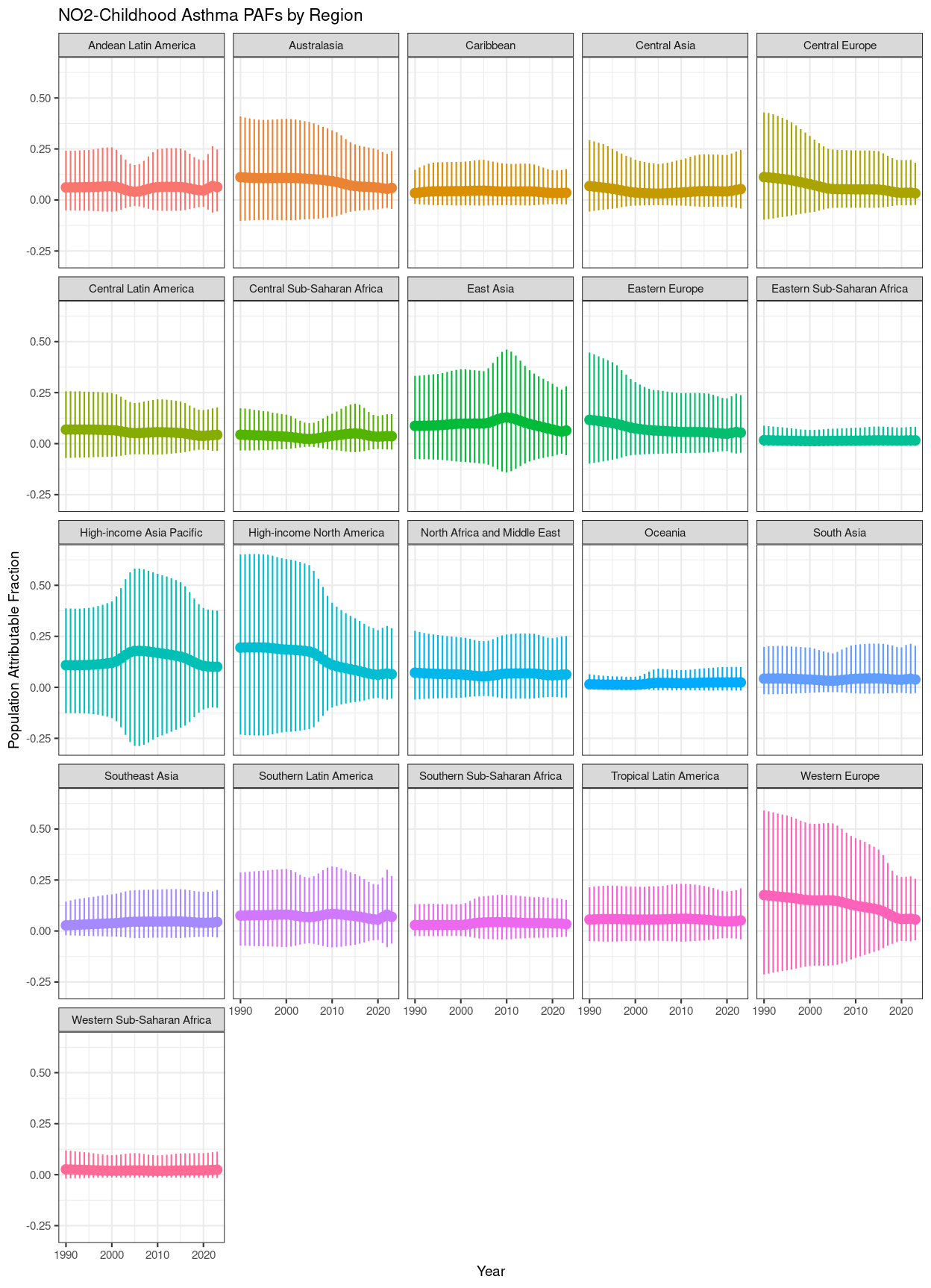


**Figure S6. Population attributable fraction of asthma YLDs attributable to** NO2 **for individuals <20 years of age by region from 1990 to 2023 with 95% uncertainty intervals**


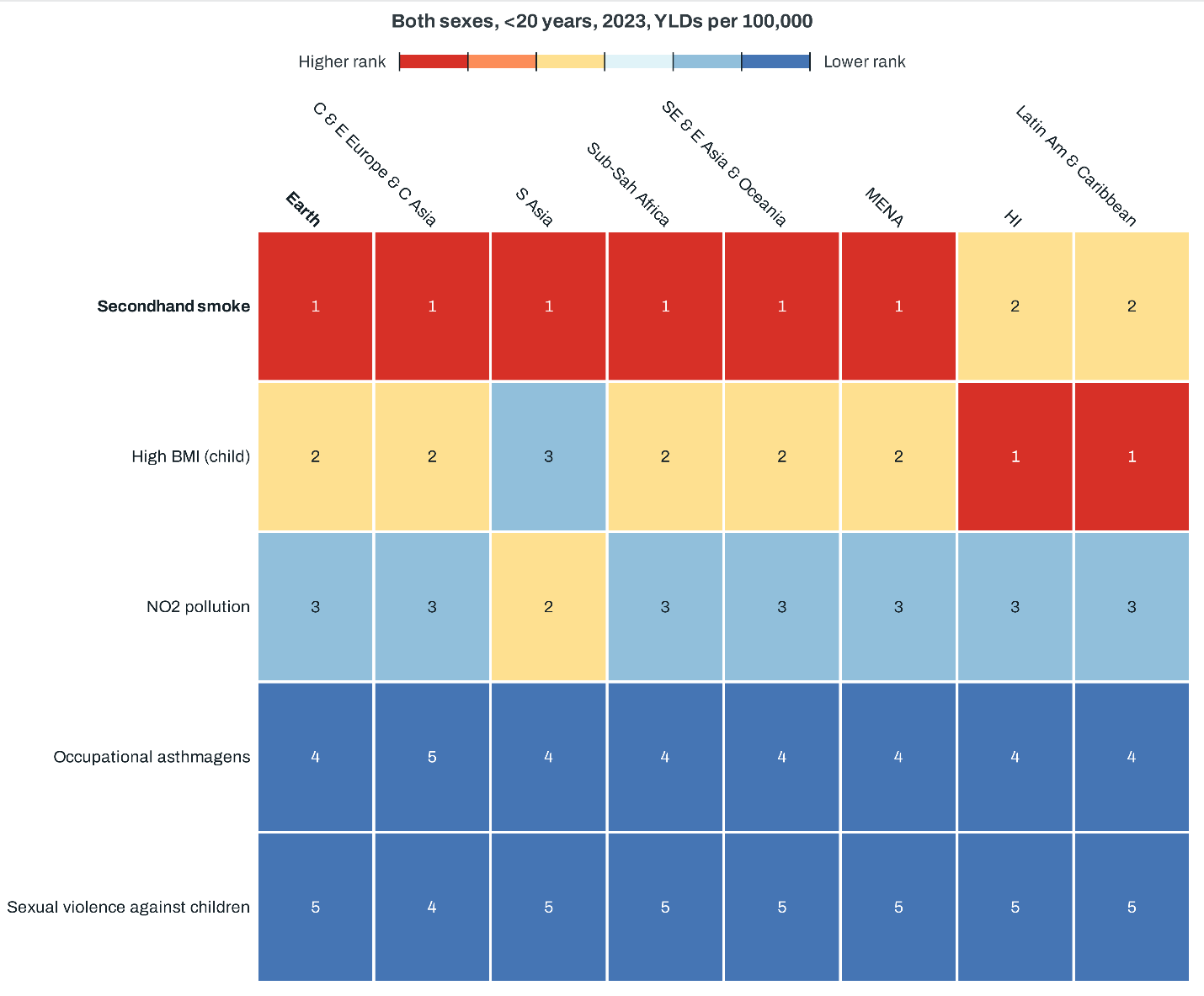


**Figure S7. Ranks of all risk factors impacting asthma YLDs in under 20 years in 2023 for all super-regions and global**


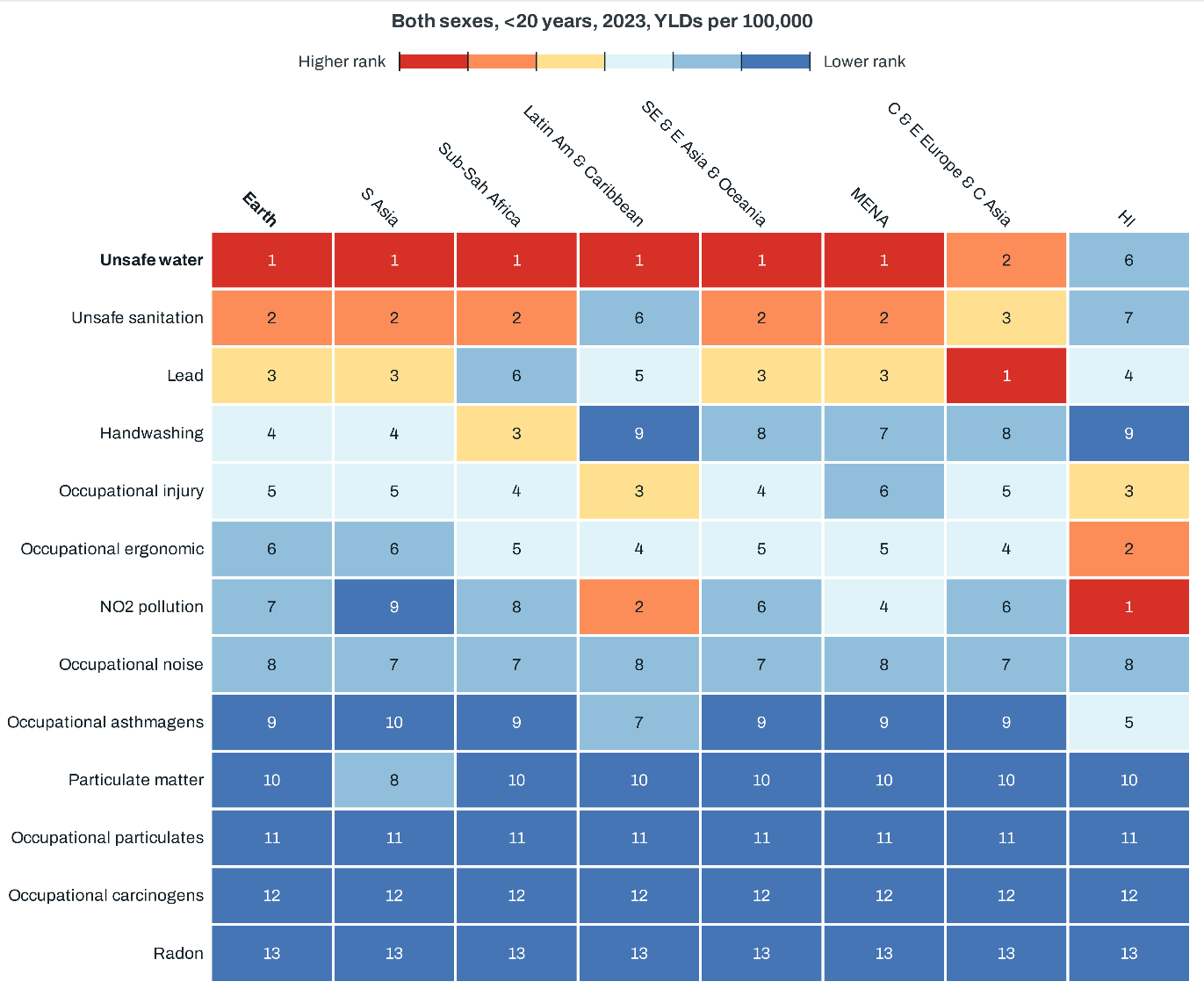


**Figure S8. Ranks of environmental/occupational risk factors impacting all YLDs in under 20 years in 2023 for all super-regions and global**

**References**

1. Khreis H, Kelly C, Tate J, Parslow R, Lucas K, Nieuwenhuijsen M. Exposure to traffic-related air pollution and risk of development of childhood asthma: A systematic review and meta-analysis. Environ Int. 2017 Mar; 100:1-31. doi: 10.1016/j.envint.2016.11.012. Epub 2016 Nov 21. PMID: 27881237.
2. Zheng P, Barber R, Sorensen R, Murray C, Aravkin A. Trimmed Constrained Mixed Effects Models: Formulations and Algorithms. J Comput Graph Stat. 2021 Jan. doi: 10.1080/10618600.2020.1868303
